# An siRNA targeting *S6k1* identifies photoreceptor phospholipid metabolism as a contributor to lipid buildup in age-related macular degeneration

**DOI:** 10.1016/j.omtn.2026.102878

**Published:** 2026-02-28

**Authors:** Shun-Yun Cheng, Delaney Giguere, San Kim, Johanna M. Seddon, Jillian Caiazzi, Katherine Gross, Nicholas McHugh, Dimas Echeverria, Julia F. Alterman, Heather Gray-Edwards, Hector Ribeiro Benatti, Lauren Renner, Hannah Woolard, Jonathan Stoddard, Trevor J. McGill, Martha Neuringer, Richard S. Brush, Martin-Paul Agbaga, Anastasia Khvorova, Claudio Punzo

**Affiliations:** 1 Department of Ophthalmology and Visual Sciences, University of Massachusetts Chan Medical School, Worcester, MA 01655, USA; 2 RNA Therapeutics Institute, University of Massachusetts Chan Medical School, Worcester, MA 01605, USA; 3 Department of Genetics and Cellular Medicine, Horae Gene Therapy Center and The Li Weibo Institute for Rare Diseases Research, University of Massachusetts Chan Medical School, Worcester, MA 01605, USA; 4 Department of Radiology, University of Massachusetts Chan Medical School, Worcester, MA 01605, USA; 5 Division of Neuroscience, Oregon National Primate Research Center, Oregon Health and Science University, Beaverton, OR 97006, USA; 6 Department of Ophthalmology, Casey Eye Institute, Oregon Health and Science University, Portland, OR 97239, USA; 7 Department of Ophthalmology and the Dean A. McGee Eye Institute, University of Oklahoma Health Sciences Center, Oklahoma City, OK 73104, USA; 8 Departments of Cell Biology, University of Oklahoma Health Sciences Center, Oklahoma City, OK 73104, USA; 9 Department of Neurobiology, University of Massachusetts Chan Medical School, Worcester, MA 01605, USA

**Keywords:** MT: Oligonucleotides: Therapies and Applications, siRNA, phospholipids, photoreceptors, Bruch’s membrane, drusen, AMD, age-related macular degeneration

## Abstract

Age-related macular degeneration (AMD) remains a leading cause for visual impairment in the elderly. We recently showed that activated mammalian target of rapamycin complex 1 (mTORC1) in photoreceptor cells causes AMD-like pathologies in mouse. Employing mouse genetics, we dissect the mTORC1 pathway and identify ribosomal protein S6 kinase beta-1 (*S6k1*) as a key component required for disease onset in our mouse model. Using a previously identified fully chemically modified tetravalent small interefing RNA (siRNA) that enriches in photoreceptors, we target *S6k1* in mouse, pigs, and non-human primates (NHP) by intravitreal injection. We find that *S6k1* silencing in diseased mice reverses phospholipid changes induced by activated mTORC1, restores lysosomal activity of retinal-pigmented epithelium cells, and reduces lipoprotein buildup at Bruch’s membrane (BM). In pigs, which do not develop disease, we find a similar shift in phospholipids as in mouse, indicating a conserved role for *S6k1* in photoreceptor phospholipid metabolism. In aged NHPs with macular drusen, the lipoprotein-rich BM deposits that are a hallmark of human AMD, *S6k1* silencing slows drusen growth over a 6-month period. These findings establish *S6k1* as modifier of lipoprotein buildup at the BM and support our siRNA platform as a potential treatment modality for AMD and other retinal diseases.

## Graphical abstract

**Figure:**
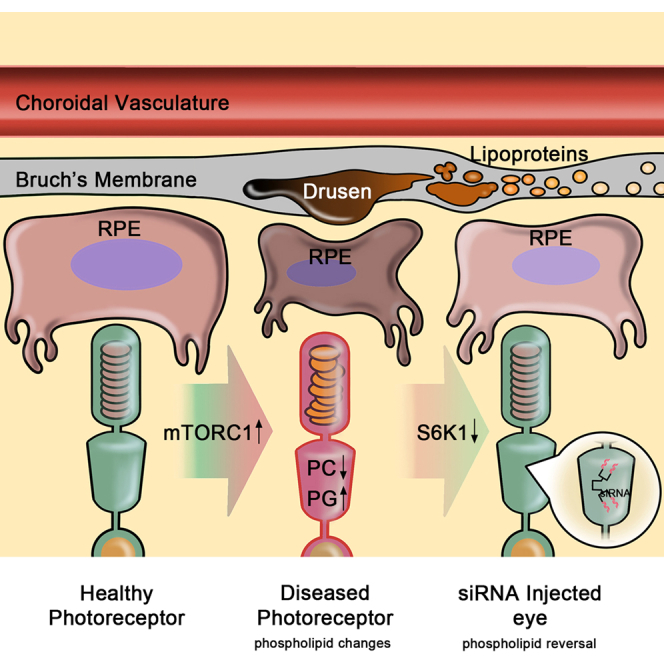

## Teaser

Punzo and colleagues show by targeting *S6k1* in mice, pigs, and non-human primates using a tetravalent siRNA that phospholipid changes in photoreceptors affect lipid buildup in the eye and progression of age-related macular degeneration. The work opens the door for the treatment of inherited blinding disorders including age-related macular degeneration.

## Introduction

Age-related macular degeneration (AMD) is traditionally considered a disease of the elderly but is increasingly being diagnosed in younger individuals. In the United States alone, approximately 18.5 million people over 40 years have early-stage AMD, and 1.5 million are affected by late-stage AMD.^1^ Approximately 20% of early-stage AMD patients will progress to the advanced disease stages of geographic atrophy (GA) and/or choroidal neovascularization (CNV), both of which can lead to severe vision loss.

Our current understanding of how AMD develops is based on the genetics of risk alleles,^2^^,^^3^^,^^4^^,^^5^ the identification of environmental risk factors,^2^^,^^4^^,^^6^^,^^7^^,^^8^^,^^9^^,^^10^ and histopathological analyses,^11^^,^^12^ which indicate that the disease initiates with the formation of a lipid wall at the choroid-retinal blood barrier, affecting primarily Bruch’s membrane (BrM) and the retinal-pigmented epithelium (RPE) basal lamina.^11^^,^^12^ Further aggregation of lipoproteins then leads to local genesis of drusen, the hallmark deposits of early AMD.^11^^,^^12^ Drusen volume and number are correlated with risk of disease progression to the advanced stages of GA and/or CNV.^13^^,^^14^^,^^15^ While it is known that oxidative stress,^16^ inflammation and complement activation,^17^^,^^18^^,^^19^^,^^20^ dysregulated lipid metabolism,^21^^,^^22^^,^^23^^,^^24^ and secretion of vascular endothelial growth factor,^25^ all contribute to the advanced disease stages, a full understanding of the underlying factors that promote disease progression remains elusive.^26^ Only non-human primates (NHPs) have a macula as in the human retina and spontaneously develop drusen. Consequently, most mouse models based on risk factors fail to recapitulate the human disease progression.^21^^,^^27^^,^^28^

We recently showed that AMD-like pathologies in mice are induced by constitutive activation of the mammalian target of rapamycin complex 1 (mTORC1)^29^^,^^30^ through deletion of one of its negative regulators, the tuberous sclerosis complex gene 1 (*Tsc1*),^31^ in rod and/or cone photoreceptor (PR) cells.^32^ These pathologies include early-stage pathologies such as the formation of a lipid wall at the BrM containing apolipoprotein B (APOB) and APOE, complement factor H (CFH) deposition, and the presence of drusen-like deposits.^32^ In contrast to these early-stage pathologies that develop uniformly in all mice, the age-dependent progression to focal RPE atrophy and neovascular pathologies (NV) occurred only in ∼20% and ∼5% of mice, respectively,^32^ which is similar to the percentages observed in humans transitioning from early-stage to advanced AMD.^1^ Finally, as in humans, we showed that disease progression is attenuated by dietary docosahexaenoic acid (DHA).^7^^,^^32^^,^^33^^,^^34^ These findings lead us to propose that metabolic adaptations in PR cells, driven by increased activity mTORC1, could contribute to disease progression in AMD.^32^

Interestingly, the phenotype that precedes any of the aforementioned pathologies in our mouse model is delayed clearance of PR outer segments (POSs) by the RPE.^32^ This phenotype correlates with a change in the composition of POS phospholipids, as measured by a reduction of di-DHA containing phospholipids.^32^ High dietary DHA supplementation was able to increase the di-DHA-containing phospholipid levels in control mice but not in mice with activated mTORC1 in rods,^32^ indicating that the changes in the POS phospholipid composition caused by activated mTORC1 recalibrate POS phospholipid levels independent of the availability of DHA. The data suggest that dysfunction of the RPE phagolysosomal pathway, which is associated with disease progression in AMD,^35^ is dependent on the phospholipid composition of the POS, which is dependent on mTORC1 activity. Interestingly, altered lipoprotein transport from the choroidal vasculature to and from PR cells, to accommodate the phospholipid needs of PR cells, is believed to contribute to lipoprotein buildup and drusen formation at the BrM.^11^^,^^12^^,^^32^^,^^36^^,^^37^

Here, we show that mTORC1 activity is increased in PR cells of AMD donor retinas at all disease stages. However, inhibiting mTORC1 activity is not a viable therapeutic strategy in humans,^38^^,^^39^ due to its role in helping PR cells adapt to the nutrient deprivation^40^^,^^41^ caused by the formation of the lipid wall at the BrM.^11^^,^^12^^,^^37^ Therefore, we genetically dissected the mTORC1 pathway to identify downstream targets that can alleviate disease caused by excessive mTORC1 activation. We find that ribosomal protein S6 kinase beta-1 (*S6k1*) is required for and has a dose-dependent effect on disease onset and progression. To study the role of *S6k1*, we employed an siRNA-mediated approach to silence *S6k1* in aged mice with early-stage AMD-like pathologies. Previously, we demonstrated that a tetravalent scaffold allows for multi-month silencing of PR-expressed genes in both mouse and pig following one intravitreal administration.^42^ Injection of a fully chemically stabilized siRNA targeting *S6k1* (tetra-siRNA^*S6k1*^) was sufficient to reduce BrM lipoprotein buildup and improve POS clearance by the RPE.

*S6k1* regulates many aspects of lipid synthesis through the sterol regulatory element-binding proteins 1 and 2 (SREBP1/2)^30^^,^^43^^,^^44^^,^^45^ and the serine/arginine-rich protein kinase-2 (SRPK2), which affect the post-transcriptional splicing of lipogenic enzymes.^46^ While mTORC1-mediated activation of S6K affects both S6K1 and S6K2, cell and lipid metabolism are mainly controlled through the mTORC1-S6K1 axis.^47^ We therefore profiled the total phospholipids of mice with and without activated mTORC1 that were injected intravitreally with the tetra-siRNA^*S6k1*^ to determine if lipid synthesis was affected by *S6k1* silencing. We found an mTORC1-dependent decrease in total PC (phosphatidylcholine) correlating with an increase in total PG (phosphatidylglycerol) phospholipids. Importantly, this shift in phospholipid classes was reversed by the tetra-siRNA^*S6k1*^ injections. Furthermore, a similar shift was found in pigs injected with the tetra-siRNA^*S6k1*^, indicating a conserved role for *S6k1* in POS phospholipid regulation. Therefore, to test if *S6k1* silencing can also affect lipoprotein buildup associated with the accumulation of the lipoprotein-rich drusen deposits, we injected two aged rhesus macaques with drusen^48^ and followed the pathology over 1 year. We found that one intravitreal injection positively affected drusen height and number over the first 6 months, slowing overall disease progression. Our studies identify *S6k1* as a modifier of POS phospholipids and lipoprotein buildup at the RPE/BrM and our tetravalent-siRNA as a safe platform for the potential treatment of retinal diseases. With a silencing effect that requires only two injections per year, the platform represents a clinically tolerable approach even for lifelong treatments.

## Results

### AMD donor retinas display increased mTORC1 activity

**Figure 1:**
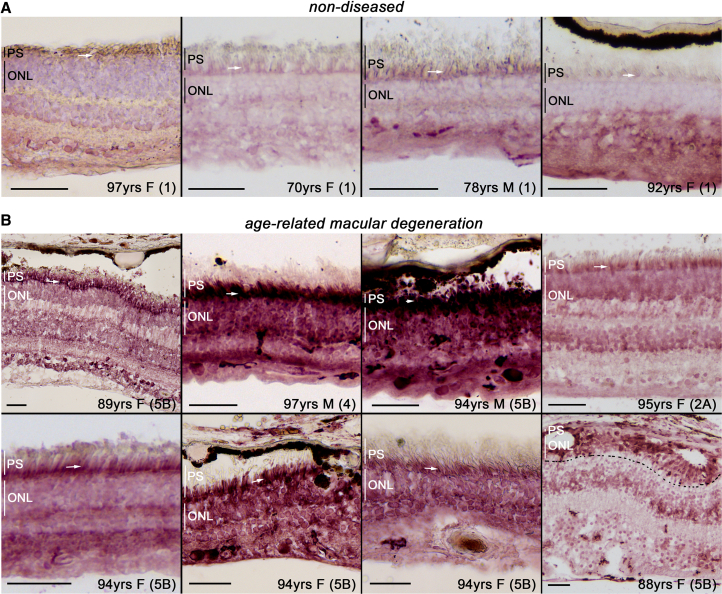
Phosphorylation of S6 is increased in retinas of AMD patients (A and B) Retinal tissue cross-sections from non-diseased donors (A) and donors with AMD (B) were immunohistochemically stained for phospho-S6 (pS6; purple signal). Higher pS6 signal is seen across all retinal layers, particularly in the PR inner segments (white arrows) and the outer nuclear layer (ONL) where PR nuclei reside. PS, PR segment region covering inner and outer segments; ONL, outer nuclear layer; vertical bars mark thickness of ONL or PS in individual images; M, male; F, female; numbers in parentheses, clinical stage (1, no disease; 2, early AMD; 3, intermediate AMD; 4, geographic atrophy; 5, neovascular AMD); dotted line in (B) demarks boundary between ONL and inner nuclear layer; Scale bars: 50 μm.

We previously showed that increasing mTORC1 activity in PR cells is sufficient to cause early- as well as late-stage AMD-like pathologies in mice.^32^ To interrogate if mTORC1 activity is increased in PR cells of AMD patients, we performed immunohistochemistry on retinal cross-sections of eight AMD donor eyes to assess the phosphorylation status of ribosomal protein S6 (pS6), a commonly used indirect readout of mTORC1 activity.^49^ When compared to four healthy donor eyes, the pS6 signal was increased in PR cells of AMD patients (Figure 1) independent of the disease stage (stages 2A–5B).^50^ The increase was seen across all retinal layers and the entire retinal section (Figure S1), with a particularly higher signal in PR inner segments and cell bodies (Figure 1).

### mTORC2 activity modulates the frequency of advanced AMD pathologies in ^*rod*^*Tsc1*^−/−^ mice

To identify a downstream mTORC1 effector that contributes to disease in mice with activated mTORC1 in rods (^*rod*^*Tsc1*^−/−^ mice),^32^ we interrupted the feedback loop between mTORC1 and mTORC2^51^ by removing mTORC2 activity through deletion of the mTORC2 accessory protein RICTOR using the Cre-lox system.^52^ At 18 months of age, mice with simultaneous deletion of *Tsc1* and *Rictor* (^*rod*^*Tsc1*^*−/− rod*^*Rictor*^−/−^) still developed focal RPE atrophy and NV pathologies (Figures S2A and S2B). In the heterozygous *Rictor* mice (^*rod*^*Tsc1*^*−/− rod*^*Rictor*^*+/−*^), the frequency of focal RPE atrophy and NV pathologies was similar to our previous data in ^*rod*^*Tsc1*^−/−^ mice,^32^ while in mice with homozygous *Rictor* deletion (^*rod*^*Tsc1*^*−/− rod*^*Rictor*^−/−^), most focal RPE atrophy observations coincided with NV pathology.

To confirm that early-stage AMD pathologies preceded advanced pathologies, we analyzed the distribution of complement component C3 and CFH as well as APOB and APOE at the RPE/BrM. ^*rod*^*Tsc1*^*−/− rod*^*Rictor*^−/−^ mice had reduced C3 expression at the RPE/BrM, which we interpreted previously as a protective response of the tissue to prevent excess complement activation^32^; however, the expression of CFH remained comparable to that of littermate controls (Figure S2C). Notably, accumulation of APOB and APOE appeared higher than what was seen in ^*rod*^*Tsc1*^−/−^ mice.^32^ Furthermore, lactate production in the retina was not significantly increased in ^*rod*^*Tsc1*^*−/− rod*^*Rictor*^−/−^ mice, confirming our previous findings that a change in lactate metabolism itself does not contribute to disease (Figure S2D).^53^ The findings suggest that mTORC2 modulates the early as well as advanced disease stages in our mouse model. The reduced accumulation of CFH and the higher accumulation of APOB and APOE could have both contributed to the higher incidence of NV pathologies seen in these mice.

### S6K1 is required for disease onset and progression in ^*rod*^*Tsc1*^−/−^ mice

**Figure 2:**
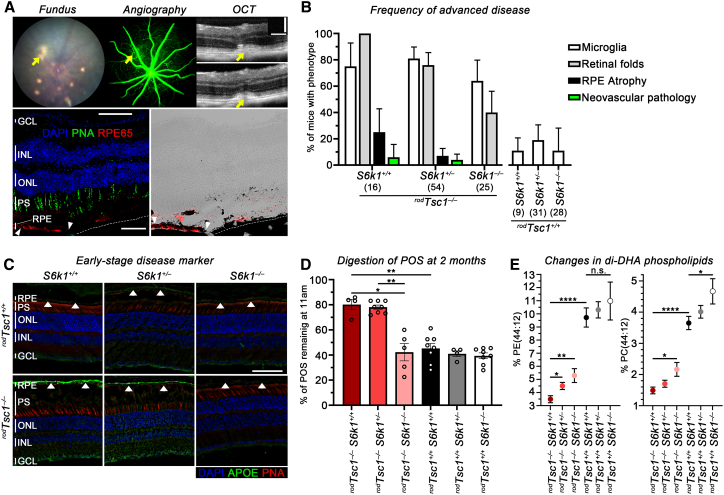
S6K1 activity is required for disease onset and progression in ^*rod*^*Tsc1*^−/−^ mice (A) Top row: representative fundus, fluorescein angiography, and OCT images of an 18-month-old ^*rod*^*Tsc1*^−/−^*S6k1*^*+/+*^ mouse with focal RPE atrophy and neovascular pathology (yellow arrows). Bottom row: immunofluorescence and bright field images with red signal from immunofluorescence staining superimposed on bright field of a retinal cross-section of the eye above (section shown in the same orientation as the OCT image) showing loss of RPE65 (red signal) expression in the area of RPE atrophy (dashed line on choroid demarks region of RPE cells loss), indicating a disrupted RPE layer. RPE65 expression with RPE cells is visible on the left third of each panel (area between white arrowheads). Only RPE atrophy is shown on section, not the neovascular pathology. Scale bars: 100 μm; blue, nuclear DAPI; green, peanut agglutinin lectin (PNA) marking cone PR segments; red, RPE65 expression marking RPE cells. (B) Frequency in percentage of phenotypes scored in each genotype at 18 months of age, including microglia activation (white bar), retinal folds (gray bars), focal RPE atrophy (black bars), and neovascular pathologies (green bars). The number of mice examined in each group is indicated in parentheses. Error bar = margin of error (M.O.E.). (C) Representative image of APOE (green signal) accumulation at the RPE/BrM (white arrowheads) in mice with indicated genotype at 12 months of age (4–5 mice were examined in each group). Scale bars: 50 μm; blue, nuclear DAPI; red, peanut agglutinin lectin (PNA) marking cone PR segments; layers in (A and C): RPE, retinal-pigmented epithelium; PS, PR segment region covering inner and outer segments; ONL, outer nuclear layer; INL, inner nuclear layer; GCL, ganglion cell layer; vertical bars in sections mark height of different layers. (D) PR outer segment (POS) clearance in RPE cells of 2-month-old mice is shown as percentage of POS remaining at 11 am when compared to 8 am in genotypes indicated (*N* = 4–8 RPE flat mounts/genotype). (E) Percentage of di-DHA PE (left) and PC (right) phospholipids as a total of PE (left) and PC (right) phospholipids in genotypes indicated (*N* = 5–6 retinas/genotype). Results in (D and E) are shown as mean ± S.E.M. (∗*p* < 0.05, ∗∗*p* < 0.01, ∗∗∗∗*p* < 0.0001; n.s., not significant).

Next, we assessed the role of *S6k1* in the pathogenesis of AMD-like phenotypes in our mice. mTORC1-mediated regulation of *S6k1* affects *de novo* lipogenesis, among other cellular pathways.^30^^,^^46^^,^^49^^,^^54^ Loss of *S6k1* (*S6k1*^−/−^) in the context of activated mTORC1 in rods (^*rod*^*Tsc1*^−/−^ *S6k1*^−/−^) prevented the development of any of the advanced AMD-like pathologies over 18 months (Figures 2A and 2B), as assessed by fundoscopy, fundus fluoresceine angiography (FFA), optical coherence tomography (OCT), and histology (Figure 2A). Allele-dependent loss of S6K1 protein was confirmed by western blotting (Figure S3). Littermate controls with two wild-type *S6k1* alleles (^*rod*^*Tsc1*^−/−^ *S6k1*^*+/+*^) developed focal RPE atrophy and NV pathologies at a similar frequency (20% and 5%, respectively) as the ^*rod*^*Tsc1*^−/−^ mice that were not crossed to the *S6k1*^−/−^ strain^32^ (Figure 2B). Heterozygous *S6k1* mice (^*rod*^*Tsc1*^−/−^ *S6k1*^*+/−*^) showed a reduction in the incidence of focal RPE atrophy and NV pathologies (7% and 2%, respectively), suggesting that the reduction of S6K1 protein levels affected the rate of progression to advanced disease (Figures 2B, S3, and S4A). Importantly, *Cre*^*–*^ control littermates with a deletion of one or two alleles of *S6k1* (^*rod*^*Tsc1*^*+/+*^ *S6k1*^*+/−*^ or ^*rod*^*Tsc1*^*+/+*^ *S6k1*^−/−^) did not display any pathologies (Figures 2B and S4A). We found that retinal lactate levels were dependent on the activation of mTORC1 and not on the loss of *S6k1*, while the increase in scotopic electroretinogram (ERG) amplitudes seen in ^*rod*^*Tsc1*^−/−^ mice compared to wild-type littermates^32^ decreased with *S6k1* loss (Figures S4B and S4C). Surprisingly, in *Cre*^*–*^ wild-type littermate controls, loss of *S6k1* led to an increase in scotopic a-wave amplitudes (Figure S4C). Although the reason for this increase is unclear, the data show that loss of *S6k1* prevents disease progression without affecting overall retinal health in ^*rod*^*Tsc1*^−/−^ mice.

To determine whether disease onset is also affected in ^*rod*^*Tsc1*^−/−^ *S6k1*^−/−^ mice, we analyzed the early-stage disease pathologies. Accumulation of APOE at the BrM was almost absent in ^*rod*^*Tsc1*^−/−^ *S6k1*^−/−^ mice (Figure 2C). Similarly, APOB and CFH protein levels were lower, while C3 was increased in ^*rod*^*Tsc1*^−/−^ *S6k1*^−/−^ mice compared to ^*rod*^*Tsc1*^−/−^ *S6k1*^*+/+*^ mice (Figure S4D), suggesting that early-stage AMD-like pathologies are being prevented in ^*rod*^*Tsc1*^−/−^ *S6k1*^−/−^ mice. Digestion of phagocytized POSs by the RPE at 2 months of age was also restored to normal in ^*rod*^*Tsc1*^−/−^ *S6k1*^−/−^ mice, with only 40% of POSs remaining by 11 am (3 h after the peak of shedding) compared to 80% of POSs remaining in ^*rod*^*Tsc1*^−/−^ *S6k1*^*+/+*^ mice (Figure 2D). Interestingly, in heterozygous ^*rod*^*Tsc1*^−/−^ *S6k1*^*+/−*^ mice, digestion of POS by the RPE was delayed to the same extend as in ^*rod*^*Tsc1*^−/−^ *S6k1*^*+/+*^ mice (Figure 2D). Consistent with this, heterozygous ^*rod*^*Tsc1*^−/−^ *S6k1*^*+/−*^ mice still progress to RPE atrophy and NV pathologies, albeit at a possibly lower rate (Figure 2B).

We previously reported that di-DHA-containing phosphatidylethanolamine (PE) and PC phospholipids (PE 44:12; PC 44:12) were significantly reduced in POSs of ^*rod*^*Tsc1*^−/−^ mice.^32^ Feeding these mice a DHA-enriched diet alleviated the AMD-like pathologies and improved POS digestion by the RPE,^32^ which correlates with many epidemiological studies linking consumption of omega-3-fatty-acid-rich diet with a risk reduction for AMD.^7^^,^^33^^,^^34^^,^^55^^,^^56^ To understand if S6K1 levels affect phospholipid metabolism in PR cells, we reprofiled these di-DHA-containing phospholipids. We found a dose-dependent increase of both di-DHA phospholipid species (PE 44:12; PC 44:12) with loss of each *S6k1* allele (Figure 2E). Although complete loss of *S6k1* did not restore the levels to the wild-type levels seen in ^*rod*^*Tsc1*^*+/+*^ *S6k1*^*+/+*^ mice, the increase occurred in both *Cre*^*–*^ and *Cre*^*+*^ mice (Figure 2E), suggesting that S6K1 protein levels affect phospholipid levels. Together, these findings suggest that removing S6K1 activity in ^*rod*^*Tsc1*^−/−^ mice prevents disease onset and progression without having overt effects on overall retinal health, while reducing S6K1 levels reduces the risk of progression to advanced disease.

### Reduction of S6K1 reverses early-stage pathologies in ^*rod*^*Tsc1*^−/−^ mice

**Figure 3:**
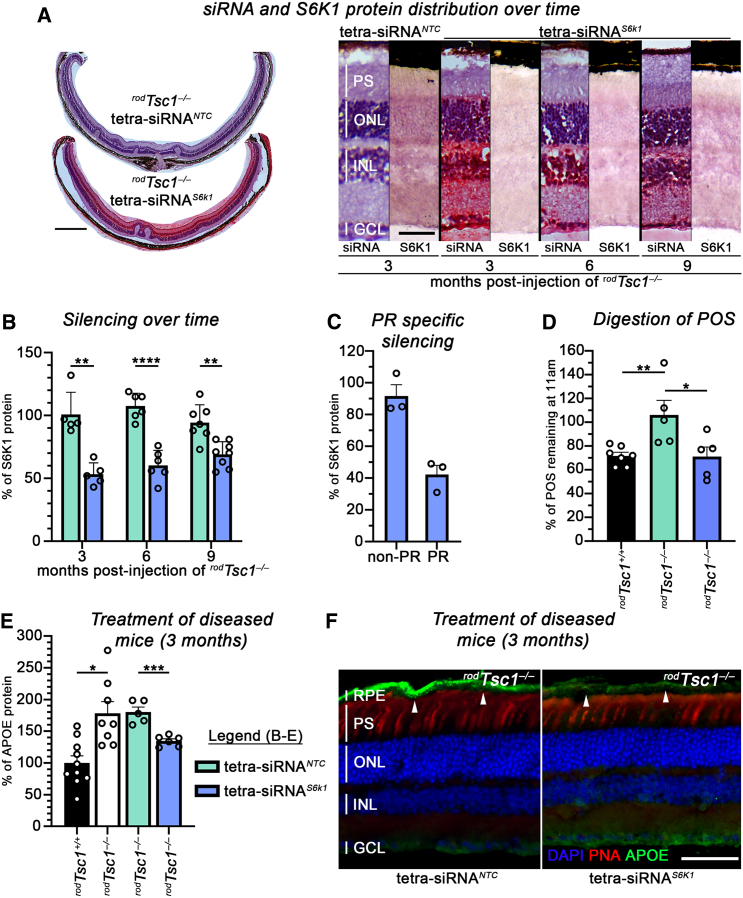
*S6k1* silencing in mouse reverses early disease pathologies in ^*rod*^*Tsc1*^−/−^ mice (A and B) Long-term siRNA retention and silencing efficacy in ^*rod*^*Tsc1*^−/−^ mice examined at 3, 6, and 9 months post-injection. Mice received one intravitreal injection of 15 μg of siRNA reagents at 3 months of age. (A) Distribution of tetra-siRNA^*S6k1*^ and S6K1 protein expression in ^*rod*^*Tsc1*^−/−^ mouse retinas. Left: tiled retinal sections showing either tetra-siRNA^*NTC*^ (top) or tetra-siRNA^*S6k1*^ (bottom, visualized with RNAScope, red signal) at 3 months post-injection (scale bars: 500 μm). Right: higher magnification of tetra-siRNA^*S6k1*^ distribution on retinal sections and S6K1 protein expression at time points indicated. Tetra-siRNA^*S6k1*^ is visualized with RNAScope (red signal), and S6K1 protein expression is visualized by immunohistochemistry (purple signal). Staining for tetra-siRNA^*S6k1*^ and S6K1 was performed on separate slides. Scale bars: 50 μm; PS, PR segment region covering inner and outer segments; ONL, outer nuclear layer; INL, inner nuclear layer; GCL, ganglion cell layer; vertical bars in sections mark height of different layers. (B) Silencing efficiency of tetra-siRNA^*S6k1*^ (blue bars) at time points indicated post-intravitreal injection when compared to the NTC (green bars). Silencing was measured by western blotting with retinal protein extracts. Mice were all injected at 3 months of age (*N* = 5–8 retinas/group). (C) Percentage silencing in PR vs. non-PR cells that were enriched by FACS at 2 months post-intravitreal delivery of siRNA. The percentage of protein expression level is normalized to the tetra-siRNA^*NTC*^ treated group. (D) PR outer segment (POS) clearance in RPE cells of 4-month-old mice shown as percentage of POS remaining at 11 am when compared to the peak of shedding at 8 am in the genotypes indicated. ^*rod*^*Tsc1*^−/−^ mice were injected at 2 months of age with siRNA reagents indicated (*N* = 4–7 eyes/group). (E and F) Reversal of APOE accumulation at the BrM in tetra-siRNA^*S6k1*^-treated mice. (E) APOE protein expression level measured by western blotting with RPE/choroid protein extracts of 15-month-old ^*rod*^*Tsc1*^−/−^ mice that are untreated or treated with either tetra-siRNA^*NTC*^ or tetra-siRNA^*S6k1*^ for 3 months (treatment started at 12 months of age). Expression levels are compared to 15-month-old littermate control ^*rod*^*Tsc1*^*+/+*^ mice (*N* = 5–10 eyes/group). (B–E) Results are shown as mean ± S.E.M. Each dot represents one retina or RPE/choroid from one mouse. Only one eye per mouse was used for each analysis (∗*p* < 0.05; ∗∗*p* < 0.01; ∗∗∗*p* < 0.001; ∗∗∗∗*p* < 0.0001; green bars represent tetra-siRNA^*NTC*^ and blue bars tetra-siRNA^*S6k1*^-injected eyes). (F) Retinal cross-section of ^*rod*^*Tsc1*^−/−^ eyes showing reduction in the accumulation APOE (green signal) at the BrM (white arrowheads) of tetra-siRNA^*S6k1*^-injected eyes (right panel). Mice were injected at 12 months of age and analyzed 3 months post-injection. Scale bars: 50 μm; blue, nuclear DAPI; red, peanut agglutinin lectin (PNA) marking cone PR segments; RPE, retinal-pigmented epithelium; PS, PR segment region covering inner and outer segments; ONL, outer nuclear layer; INL, inner nuclear layer; GCL, ganglion cell layer; vertical bars in sections mark height of different layers.

To determine the effect of reducing S6K1 levels after symptoms develop, we used an siRNA approach to target *S6k1* in 1-year-old ^*rod*^*Tsc1*^−/−^ mice. We screened 21 sequences that were predicted by an algorithm developed by the Khvorova laboratory^57^ and identified one sequence with strong *S6k1* silencing *in vitro*, which is conserved among mammals, including mouse, swine, NHP, and human genomes (Figure S5). Using the tetrameric siRNA configuration, which we have previously identified as being efficient at entering PR cells,^42^ we tested the *S6k1*-targeting sequence (tetra-siRNA^*S6k1*^) for safety and silencing efficiency in an initial dose-response study in mice (Figure S6). Gene silencing plateaued at a reduction of approximately 50% of S6K1 protein levels with a dose of 15 μg or higher, while no toxicity was seen even at the highest dose of 50 μg. To determine the longevity of silencing, we delivered 15 μg of tetra-siRNA^*S6k1*^ in 3-months-old ^*rod*^*Tsc1*^−/−^ mice and analyzed retinal tissue at 3, 6, and 9 months post-injection by RNAscope, immunohistochemistry, and western blotting (Figures 3A and 3B). Similar to the dose-response, S6K1 levels were reduced by ∼50% at 3 months post-injection and ∼40% and ∼30% at 6 and 9 months post-injection, respectively. To determine the silencing efficiency in PR cells, we used fluorescence-activated cell sorting (FACS) of tdTomato-labeled PR cells (Figure S7) harvested from mice injected with the tetra-siRNA^*S6k1*^ or the non-targeting control (NTC: tetra-siRNA^*NTC*^)*.* We found a 60% reduction of S6K1 protein levels in PR cells compared to a 10% reduction in non-PR cells (Figure 3C), indicating that the tetra-siRNA^*S6k1*^ achieves a stronger reduction of S6K1 in rod PR cells when compared to the reduction seen with removal of one *S6k1* allele (^*rod*^*Tsc1*^−/−^ *S6k1*^*+/+*^ versus ^*rod*^*Tsc1*^−/−^ *S6k1*^*−/+*^), which resulted in a 36% drop in S6K1 protein (Figure S3A). Consistent with this, injection of the tetra-siRNA^*S6k1*^ in 2-month-old ^*rod*^*Tsc1*^−/−^ mice was able to restore POSs clearance within a 2-month time window when compared to the tetra-siRNA^*NTC*^-injected mice (Figure 3D). The 2-month time window was chosen to allow enough time for silencing to reach peak levels, for the changes in phospholipids to be reflected in the POS, and for any effect on the RPE phagolysosomal pathway to take hold. To test if this improvement in RPE health affects early-stage pathologies such as lipoprotein buildup at the RPE/BrM, we injected 1-year-old ^*rod*^*Tsc1*^−/−^ mice, where we found a significant decrease in APOE at the RPE/BrM over a 3-month period (Figures 3E and 3F). Clinical grading of immunofluorescence stainings for the other early AMD markers on tissue sections showed the same trend, with decreases in APOB and CFH levels and an increase in C3 (Figure S8). Together, the data show that a ∼50% reduction of S6K1 protein in PR cells of ^*rod*^*Tsc1*^−/−^ mice by the tetra-siRNA^*S6k1*^ is sufficient to restore RPE phagolysosomal activity, to reduce buildup of lipoproteins at the RPE/BrM and of CFH, while starting to restore normal C3 expression, suggesting that overall RPE health is being improved.

### Effect of tetra-siRNA^*S6k1*^ on phospholipids is conserved between mouse and pig

**Figure 4:**
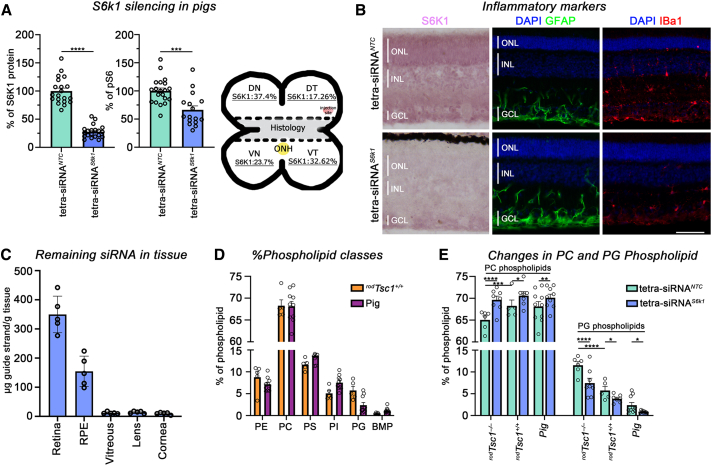
Silencing of *S6k1* in pigs (A) Bar graphs from western blot quantifications showing a 72% reduction in S6K1 protein levels and a 34% reduction in pS6 protein levels when comparing the tetra-siRNA^*S6k1*^-injected eyes to the NTC-injected eyes at 3 months post-intravitreal injection of 300 μg in 100 μL volume of siRNA reagent. A total of five pigs were used, each having one eye injected with the tetra-siRNA^*S6k1*^ and the other with the tetra-siRNA^*NTC*^. Eyes were dissected in four quadrants with the central portion used for histology (see sketch in A) and the quadrants used for quantification of silencing efficiency. Each dot in the bar graphs represents one quadrant from one animal (error bars = ± S.E.M.; ∗∗∗*p* < 0.001, ∗∗∗∗*p* < 0.0001). The sketch details the silencing efficiency within each quadrant. S6K1 silencing was most efficient close to the injection site (marked in red in sketch; D, dorsal; V, ventral; T, temporal; N, nasal). (B) Representative antibody staining for S6K1 (first column, purple signal), GFAP (second column, green signal), and Iba1 (third column, red signal) on retinal cross-sections at 3 months post-intravitreal injection of 300 μg of each siRNA reagent. Sections originated from the central strip of the retina (see sketch in A). Blue, nuclear DAPI; green, GFAP; red Iba1; Scale bars: 100 μm; ONL, outer nuclear layer; INL, inner nuclear layer; GCL, ganglion cell layer; vertical bars in section mark height of different layers. (C) Pharmacokinetics of remaining siRNA in different eye tissues at 3 months post-injection. A PNA hybridization assay was performed with one quadrant per eye (*N* = 5, error bars = ± S.E.M.). Analyses of the retina, RPE, vitreous, lens, and cornea show that most of the siRNA is taken up by the retina and RPE and is cleared from the vitreous, with little migrating to the lens and cornea. Eye tissues from NTC-injected eyes did not show any signal and were therefore omitted from the figure. (D) Total phospholipid breakdown by class of phospholipid [PE: phosphatidylethanolamine; PC: phosphatidylcholine; PS: phosphatidylserine; PI: phosphatidylinositol; PG: phosphatidylglycerol, BMP: bis(monoacylglycero)phosphate]. Shown is %-distribution of each class of phospholipid listed for wild-type mouse (^*rod*^*Tsc1*^*+/+*^) and pig retinas with the sum of them representing 100%. (E) Changes in PC and PG phospholipids in retinas of ^*rod*^*Tsc1*^−/−^ mice, ^*rod*^*Tsc1*^*+/+*^ mice, and pigs injected intravitreally with the tetra-siRNA^*NTC*^ or the tetra-siRNA^*S6k1*^. Error bars in (D and E) show S.E.M.; ∗*p* < 0.05, ∗∗*p* < 0.01, ∗∗∗*p* < 0.001, ∗∗∗∗*p* < 0.0001; *N* = 5–6 mouse retinas and 8–10 pig retinal samples.

POS membrane discs that are daily phagocytosed by the RPE are highly enriched in phospholipids. The RPE digests and metabolizes some of the fatty acids, while others are recycled back to PR cells or released into the circulation as apolipoprotein particles. New lipoprotein particles from the circulation are also taken up to help maintain the phospholipid synthesis needs of PR cells due to the shedding of the POS. Dysregulation of these processes is believed to contribute to lipoprotein buildup and drusen formation at the RPE/BrM.^11^^,^^12^^,^^36^^,^^37^ Since the tetra-siRNA^*S6k1*^ was able to restore RPE phagolysosomal activity and reduce lipid buildup at the BrM of ^*rod*^*Tsc1*^−/−^ mice, we performed a more in-depth phospholipid analysis to determine which phospholipid classes are changing as a function of mTORC1 activation and *S6k1* reduction and if such changes are conserved across species. To that end, we used retinas of pigs injected intravitreally with the tetra-siRNA^*S6k1*^ at a dose of 300 μg/eye, which we previously established to be safe and efficacious.^42^ We found a 72% reduction in S6K1 protein and a 34% decrease in pS6 levels at 3 months post-injection (Figure 4A). Histological analysis indicated that retinas injected with tetra-siRNA^*S6k1*^ exhibited reduced S6K1 signal across the retinal section with no signs of severe gliosis or microglia activation, mirroring the previous data for silencing of the *Huntingtin* (*HTT*) gene^42^ (Figure 4B). Pharmacokinetics analyses using a peptide nucleic acid (PNA) hybridization assay^58^ to determine the distribution of the siRNA in different tissues of the eye at 3 months post-injection revealed that most siRNAs were in the retina followed by the RPE, with little to nothing remaining in the vitreous or having migrated to the lens and cornea (Figure 4C). Finally, comprehensive lipidomics analyses of PE, PC, PS (phosphatidylserine), PI (phosphatidylinositol), PG, and BMP [bis(monoacylglycero)phosphate] phospholipids revealed similar levels of these phospholipids between mouse and pig (Figure 4D). Among these phospholipids, only total PC and total PG showed a significant change in their percentile representation between the tetra-siRNA^*NTC*^- and tetra-siRNA^*S6k1*^-injected eyes (Figure 4E). Specifically, *S6k1* silencing induced the same directional change in both pig and mouse retina, where total PC increased with a corresponding decrease in total PG. This directional change in total PC and total PG counteracted the effect of activated mTORC1 in ^*rod*^*Tsc1*^−/−^ mice, which led to a decrease in total PC and an increase in total PG. The data indicate that the changes caused by *S6k1* silencing are similar between mouse and pig and that the effects on phospholipid synthesis caused by constitutively activated mTORC1 in rods are partially mitigated by the reduction in *S6k1* expression.

### Tetra-siRNA^*S6k1*^ affects drusen in NHPs

The findings in mouse and pig prompted us to test if the tetra-siRNA^*S6k1*^ can affect lipoprotein buildup in a more natural model of the disease, such as aged rhesus macaques, which spontaneously develop lipoprotein-rich drusen deposits.^48^ To test this, we first injected the left eye (oculus sinister; OS) of a young rhesus macaque with a dose of 225 μg of tetra-siRNA^*S6k1*^ while using the contralateral right eye (oculus dexter; OD) as an uninjected control to test silencing and safety. At 1 month post-injection, we found uniform silencing of *S6k1* on retinal sections across the entire temporal-nasal axis, with a ∼50% reduction in S6K1 protein levels by western blot, indicating that the therapeutic level of silencing established in our mouse model is achieved with this dose in an NHP (Figures S9A and S9B). Antibody staining and western blotting for pS6 confirmed the reduction in S6K1 levels (Figures S9C and S9D); in particular, reduction of pS6 in PR inner segments was evident by antibody staining (Figure S9C, arrows), while staining for the microglia marker Iba1, gliosis marker glial fibrillary acidic protein (GFAP), and medium wavelength opsin (Figures S10A–S10C) showed that the siRNA, the distribution of which was visualized by RNAscope (Figure S10D), had no negative impact on retinal health.

**Figure 5:**
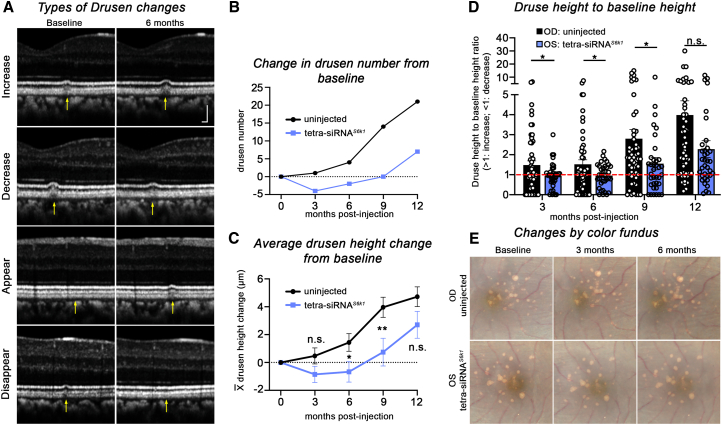
Injection of the tetra-siRNA^*S6k1*^ halts drusen progression in NHP (A) Example of drusen changes seen by OCT between baseline and 6 months post-intravitreal injection of the tetra-siRNA^*S6k1*^. “Increase” and “Appear” show examples from uninjected (OD) eyes while “Decrease” and “Disappear” from injected (OS) eye (changes are marked by yellow arrows). Scale bars: 100 μm. (B) Change in drusen number between injected and uninjected eyes. At baseline there were 27 drusen between the two uninjected eyes and 25 between the two injected eyes. (C) Average change in drusen height in μm plotted as the absolute change from baseline over time (data show mean ± S.E.M.; ∗*p* < 0.05; ∗∗*p* < 0.01; n.s., not significant). (D) Ratios of drusen height at time point indicated relative to drusen height at baseline. Each dot represents an individual druse. Ratio >1 indicates a druse that grew larger (red dotted line: ratio = 1; bars show mean of ratios ± S.E.M.; ∗*p* < 0.05; n.s., not significant). All measurements shown in (B–D) are reflective of changes seen within the central region of the eye that was scanned (see also Figure S11). (E) Color fundus photographs of the central fundus of the 22-year-old rhesus macaque injected with the tetra-siRNA^*S6k1*^ in its left eye (OS), showing a slight improvement of pathologies from baseline at 3 and 6 months compared to the untreated eye (OD), where pathologies appear to worsen over time (OD: right eye, uninjected; OS: left eye, injected).

Next, two aged rhesus macaques (21 and 22 years old, one male and one female) with multiple large macular drusen were injected intravitreally with 225 μg of the tetra-siRNA^*S6k1*^ in their left eyes (OS) while keeping the right eyes (OD) as uninjected controls. The study was non-terminal due to the limited availability of aged NHPs with drusen. Multimodal retinal imaging was conducted at 3-month intervals for a total of 1 year, allowing us to track changes in drusen number and height over time (Figure S11). In addition, because small drusen can appear and disappear (Figure 5A), we tracked the behavior of each druse over time within the scanned area and calculated change ratios, defined as druse height at the time of analysis divided by its baseline height at the time of injection. This allowed us to determine whether a druse was increasing in height (ratio>1), decreasing (ratio<1), or disappearing (ratio = 0).

Analyses of OCT images showed a decrease in the total drusen number from 25 at baseline for both injected eyes to 21 and 23 at 3 and 6 months post-injection, respectively. The number returned to baseline by 9 months and climbed to 32 by 12 months post-injection. In the uninjected OD eyes, the total number of drusen continuously increased at every time point from 27 at baseline to 48 by 12 months post-injection (Figure 5B). Interestingly, the increase in drusen number between 9 and 12 months was the same in the injected (OS) and uninjected (OD) eyes (seven drusen). This suggests that the initial reduction in number during the first 6 months was likely caused by the tetra-siRNA^*S6k*^. Similarly, the average drusen height decreased from baseline at 3 and 6 months post-injection and then slightly increased above baseline by 9 months post-injection in the injected eyes. In the uninjected eyes, the average drusen height increased continuously during the entire study (Figure 5C). Plotting the change ratios of all drusen over time (Figure 5D) showed that most drusen in the tetra-siRNA^*S6k1*^ injected eyes (OS) had a ratio around 1 at 3 and 6 months post-injection, with the average of the ratios being slightly below 1 at both time points, whereas averages were above 1 for the uninjected eyes. The differences seen in drusen number, heights, and ratios of heights between injected and uninjected eyes suggest that overall progression of drusen growth was reduced in the injected eyes over a period of 6 months. The findings suggest that as in mouse (Figures 3E and 3F), lipoprotein accumulation at the RPE/BrM that contributes to drusen growth may be reduced or slowed over the silencing period (Figure 3B). Color fundus photographs of the macular area at baseline, 3 months, and 6 months post-injection further support the conclusions from the OCT analyses, showing a slight alleviation of pathologies in the injected OS eyes during the first 6 months of the observation period when compared to the uninjected OD eyes, where pathologies progressively worsened (Figures 5E and S12). Furthermore, while pathologies worsened in both eyes by 12 months post-injection, the overall changes seen by fundus photography appear less pronounced in the injected eyes when compared to the uninjected eyes (Figure S13). In summary, the data suggest that one intravitreal injection of the tetravalent siRNA^*S6k1*^ is sufficient to affect drusen growth for a period of 6 months. Furthermore, it suggests that safe and effective gene silencing with the tetrameric configuration can be maintained with two intravitreal injections per year, opening possible new treatment opportunities for many retinal degenerative diseases.

## Discussion

In this study, we show that *S6k1* activity in PR cells, driven by increased mTORC1 activity, affects the phospholipid composition of POSs, which in turn affects the RPE phagolysosomal pathway, causing delayed POS clearance in RPE cells and lipid buildup at the BrM. How do our findings align with our current understanding of AMD disease progression? Age, lifestyle, and genetic risk factors contribute to atherosclerotic conditions that eventually manifest in choriocapillaris impairment in the macular area, resulting in a delayed rod-mediated dark adaptation.^59^^,^^60^^,^^61^ Interestingly, in patients with late-stage advanced disease, ophthalmic artery angioplasty improves perceived visual function.^62^^,^^63^ The formation of a lipid wall at the RPE/BrM further compounds the problem of nutrient flow from the choroidal vasculature to RPE and PR cells, since the presence of a hydrophobic lipid wall functions as a natural barrier for hydrophilic molecules such as glucose.^11^^,^^12^ Here, we propose that once nutrient supply from the choroidal vasculature to RPE and PR cells is reduced below a certain threshold due to the thickening of the lipid wall at the RPE/BrM and the atherosclerotic condition that reduces choriocapillaris blood flow, there is an adaptive change in PR cells that leads to an increase in mTORC1 activity to help render PR cells more resilient to the nutrient deprivation experienced.^64^^,^^65^ In line with this idea of an adaptive response of the whole retina, a recent quantitative trait locus (QTL) mapping analysis found significant changes in the methylation patterns of genes related to cellular metabolism and mTOR signaling in the retinas of patients with AMD at all disease stages.^66^ The nutrient problem may be exacerbated even more due to the metabolic coupling of PR and RPE cells. Under normal circumstances, the RPE uses lactate secreted by PR cells and fatty acids derived from shed PR outer segments (POSs) as an energy source.^26^^,^^67^^,^^68^^,^^69^ However, a shortage of glucose in PR cells reduces the amount of lactate and fatty acids PR cells provide to the RPE, causing RPE cells to consume glucose themselves rather than transporting it to PR cells.^26^^,^^67^^,^^68^^,^^69^ In contrast to the human disease, pathological changes in our mouse model are not preceded by the development of atherosclerosis and the formation of a lipid wall at the BrM; rather, they start directly with the mTORC1-driven adaptive response in PR cells.^32^ The model shows that this response is sufficient to contribute to lipoprotein buildup at the BrM, drusen genesis, and over time, to focal RPE atrophy and NV pathology.^32^ Increasing mTORC1 activity in PR cells also affects the composition of POS phospholipids, a change that attenuates RPE phagolysosomal activity. Since RPE lysosomal dysfunction has been associated with drusen formation, lipofuscin accumulation, increased oxidative stress, and disease progression in AMD,^35^^,^^70^^,^^71^^,^^72^ our model aligns in many respects with the current knowledge of disease progression in AMD.

The concept that PR cell metabolism contributes to AMD has been introduced by several human studies.^11^^,^^12^^,^^73^^,^^74^^,^^75^ Analyses of the distribution of soft drusen and subretinal drusen deposits, both lipoprotein-rich deposits of the early disease stage, revealed that their preferential locations mirror the densities of cones and rods, respectively.^11^^,^^12^^,^^75^ Consistent with these observations, soft drusen-like deposits in our mouse model required activation of mTORC1 in cones.^32^ Macular translocation procedures, used in patients with GA to save foveal cones from an underlying dying RPE, resulted in redevelopment of GA where the macular cones were translocated.^73^^,^^74^ These and other findings led to the proposal that the high and different metabolic demands of cones and rods are what contributes to the early- as well as late-stage pathologies.^11^^,^^12^^,^^73^^,^^74^^,^^75^ However, only ∼10% of adults over 50 years of age develop AMD,^1^ which led us to investigate whether PR cell metabolism is intrinsically different in patients with AMD.^32^ Similar to what we saw in nutrient-deprived cones of mice with retinitis pigmentosa,^64^ we found increased mTORC1 activity is in PR cells of AMD patients regardless of the disease stage, as well as increased expression of glycolytic genes.^32^ The broad increase in pS6 signal in humans is indicative of a retinal and PR adaptive response, which aligns with the recent QTL mapping analysis finding significant changes in the methylation patterns of genes related to cellular metabolism and mTOR signaling in the retinas of patients with AMD.^66^ Furthermore, we showed in our previous study that increasing mTORC1 activity in PR cells is sufficient to induce AMD-like pathologies, including lipoprotein and CFH buildup at the BrM, drusen driven by cones, focal RPE atrophy, and NV pathologies resembling GA and CNV in humans, respectively.^32^ Here, we show that this increase leads to *S6k1*-dependent POS phospholipid changes and that when reversed, restores RPE phagolysosomal activity and prevents lipoprotein buildup at the RPE/BrM. Indeed, targeting the RPE phagolysosomal pathway, in particular transcription factor EB (TFEB), is seen as a therapeutic avenue for AMD.^35^^,^^71^^,^^76^

S6K1 regulates fundamental cellular processes including protein and lipid synthesis mediated by the upstream kinase mTORC1.^45^^,^^49^ Our results suggest that S6K1-mediated phospholipid synthesis may play a significant role in the pathology of the disease. The decrease in total PC phospholipids and the increase in total PG phospholipids mediated by activated mTOCR1 in ^*rod*^*Tsc1*^−/−^ mice were counteracted by the tetra-siRNA^*S6k1*^ in mouse. Since PG is used to synthesize PC and PE, the data support the idea of dysregulated retinal lipid metabolism as a function of activating the mTORC1-S6K1 axis. While the other phospholipid classes did not show a significant change in their total percentages, individual phospholipids within the other classes did change, such as the di-DHA PE species.^32^ It remains unclear which phospholipid changes affect RPE phagolysosomal activity and contribute to disease onset and progression. Perturbations in the intracellular ratios of phospholipids and omega-3 fatty acids have been associated with defects in autophagy and lysosomal function.^77^^,^^78^^,^^79^^,^^80^^,^^81^ Thus, changing the composition of the POS would inevitably change the RPE intra-lysosomal as well as intracellular composition of phospholipids and fatty acids. Our data suggest that the phospholipid composition of the POS may be on a continuum that depends on the level of S6K1 activity. This in turn may affect the severity and speed of disease progression. For example, heterozygous *S6k1* mice (^*rod*^*Tsc1*^−/−^ *S6k1*^*−/+*^) still displayed delayed POS clearance and progression to advanced disease, albeit the percentage of mice with advanced disease is lower, in contrast to the tetra-siRNA^*S6k1*^-injected mice, which had a slightly greater percentage in S6K1 protein reduction and normal POS clearance. It remains to be determined whether the tetra-siRNA^*S6k1*^ is able to prevent progression to the advanced pathologies in ^*rod*^*Tsc1*^−/−^ mice, nor do we know the POS phospholipid composition of heterozygous *S6k1* (^*rod*^*Tsc1*^−/−^ *S6k1*^*−/+*^) mice. Interestingly, analyses of phospholipids in human blood samples of AMD patients identified alterations in metabolites involved in glycerophospholipid metabolism,^82^^,^^83^ supporting a role for phospholipid metabolism in AMD pathogenesis. In addition, previous studies identified AMD-related genes in the high-density lipoprotein (HDL) pathway, including *LIPC*.^23^^,^^24^ It is also notable that serum protein analyses identified S6K1 as one of 28 proteins enriched in serum of AMD patients.^84^ Components of PR cells or autoantibodies to such components are often seen in the serum of individuals with PR cell degeneration. Autoantibodies to pyruvate kinase muscle isozyme M2 (*PKM2*), a gene we have previously shown to be upregulated in PR cells of AMD patients,^32^ have been associated with AMD independent of the disease stage.^85^

Our findings suggest that the mTORC1-S6K1 signaling axis plays a critical role in AMD pathogenesis, as it correlates with many human findings that support the notion of dysregulated glycerophospholipid synthesis as a contributor to AMD. Here, we correlated *S6k1*-regulated POS phospholipids to RPE-phagolysosomal activity and lipoprotein buildup at the RPE/BrM. Phagolysosomal activity of the RPE has been linked by other studies to lipoprotein buildup and disease progression in AMD.^35^^,^^70^^,^^71^^,^^72^^,^^76^ We found that regulation of POS phospholipids by *S6k1* was similar between pig and mouse and that NHPs with drusen injected with the tetra-siRNA^*S6k1*^ displayed alleviated pathologies. This suggests a conserved role for *S6k1* in POS phospholipid synthesis. While our results in the NHP need to be interpreted with caution due to the limited number of animals used, the data align with all other findings in mouse, including the duration of silencing being between 6 and 9 months. Importantly, the tetrameric siRNA configuration used here in NHPs for silencing of *S6k1* can be used safely for the silencing of many other retinal disease genes that cause blindness with a treatment regimen of two intravitreal injections per year. Because correcting RPE phagolysosomal dysfunction through TFEB is a therapeutic avenue being considered, it is interesting to speculate if correcting it by altering what the RPE “eats” might not be a better therapeutic approach,^35^^,^^71^^,^^76^ as the latter treats the cause and not a symptom. Additionally, *S6k1* may also have a more pleiotropic effect on disease progression. Constitutive activation of mTORC1 in the RPE has been shown to affect RPE and PR health^86^^,^^87^ and contribute to RPE senescence,^88^ while S6K1 has been shown to regulate the inflammatory senescence-associated secretory phenotype.^89^ Thus, it is possible that the tetra-siRNA^*S6k1*^ may also have a direct positive effect on the RPE.

In summary, the data presented in this study suggest that adaptations to nutrient stress in PR cells triggered by atherosclerotic conditions and lipid buildup at the BrM lead to a permanent metabolic shift in PR cells that is governed by the kinase mTOR and manifests in part as a change of POS phospholipid composition. We identify *S6k1* as a modifier of POS phospholipids, RPE phagolysosomal activity, and lipoprotein buildup at the RPE/BrM. Furthermore, we show that the tetrameric siRNA configuration is a safe therapeutic reagent for gene silencing in the retina.

## Materials and methods

### Sex as a biological variable

No sex-specific differences were observed with regard to the pathologies presented in this study. Therefore, sex was not considered as a biological variable.

### Human donor eyes

Donor tissues were obtained from the Eye Donation Project^90^ under the protocol of J.M.S. that was approved by the Institutional Review Board of the University of Massachusetts Chan Medical School. Human retinal tissues were obtained from patient donors with and without AMD after obtaining informed consent. The clinical diagnosis and severity of AMD were determined by reviewing and grading ocular examination records and multimodal imaging of eye donors in the Seddon Longitudinal Cohort by J.M.S. Immunohistochemistry analysis was done on retinal cross-sections of 12 human donor eyes (age, severity of AMD, and gender are indicated in figure legends). Four eyes had no clinical evidence of AMD, and eight eyes had different severities of AMD (2A: drusen deposits, 4: GA, and 5B: CNV). The time from death to enucleation ranged from 2 to 9 h.

### Animal models

All procedures involving animals were in compliance with the Association for Research in Vision and Ophthalmology (ARVO) Statement for the Use of Animals in Ophthalmic and Vision Research. All mouse studies, pig studies, and the young rhesus macaque study were approved by the Institutional Animal Care and Use Committees (IACUC) of the University of Massachusetts Chan Medical School and were conducted at the University of Massachusetts Chan Medical School; studies of aging macaques conducted at the Oregon National Primate Research Center (ONPRC) were approved by the IACUC of Oregon Health & Science University. The conditional *Tsc1*,^31^ *Rictor*,^91^ and the *S6k1*^−/−^^92^ mice as well as the rod *iCre-75*^93^ have all been previously described. A summary of the genotypes generated, the mouse models used, and experiments performed is presented in Table S1 of the supplemental information file. All mice were genotyped for the absence of the *rd8* mutation.^94^ Mice were kept on a 12-h light/12-h dark cycle with unrestricted access to food and water. Equal numbers of male and female mice were used in all experiments. No sex-specific differences were noted. All experiments compared indicated genotypes with littermate controls originating from heterozygous crosses. The pigs used in the study were between the ages of 1 and 2 months at the time of injection and were a Yorkshire breed purchased from Earl Parsons and Sons. The young rhesus macaque was purchased from Worldwide Primates Inc. The two aged rhesus macaques with significant drusen accumulation (one male, one female, 21 and 22 years old at study initiation) were studied at ONRPC. All animals, mouse, pig, and rhesus macaques were fed standard diets.

### Intravitreal injection of siRNA

Intravitreal injections in mouse were performed as previously described.^95^ Glass needles (Clunbury Scientific LLC; Cat no. B100-58-50) were used in combination with the FemtoJet (Eppendorf) with a constant pressure and time (300 psi and 1.5 s, respectively) to deliver ∼2 μL of siRNA into the vitreous. The initial dose-response experiment used various amounts of siRNA, but all subsequent experiments used 15 μg/eye in mouse. Intravitreal injections in pigs were done under anesthesia, which was performed and supervised by animal medicine of the University of Massachusetts Chan Medical School. After pigs were fully anesthetized, proparacaine and iodine were used to numb and disinfect the eye, respectively. A sterile insulin syringe was used to deliver 100 μL of siRNA into each eye (300 μg/eye). The injection sites were always ∼2–3 mm away from the limbus on the temporal side of the eye. After injection, sterile saline was used to rinse the exterior of the eye. Intravitreal injections were performed in a young rhesus macaque at the University of Massachusetts Chan Medical School and in two aged rhesus macaques at ONPRC similarly to the injection in the pig eyes using a volume of 75 μL and a total dose of 225 μg. At ONRPC, the intravitreal injection was followed by subconjunctival injection of steroid (10 mg dexamethasone) and antibiotic (125 mg cefuroxime) and topical application of erythromycin ointment. All intravitreal injections used the same conserved tetrameric siRNA sequence shown in Figure S5. Both siRNA reagents, the tetra-siRNA^*S6k1*^ and the tetra-siRNA^*NTC*^, were synthesized once as two large individual batches, and all injections were done with the same batch. In all experiments except for the rhesus macaques, one eye was injected with the tetra-siRNA^*S6k1*^ and the contralateral eye as a control with the tetra-siRNA^*NTC*^. The reason for not using an NTC for the aged rhesus macaque study on animals with drusen was the limited safety data in this species, the circumstance that the study was non-terminal and that animals with a pathology were being injected. To avoid damaging both eyes in case of unexpected excessive inflammation due to the tetrameric siRNA as a reagent in NHPs with drusen, we opted to leave one eye as untreated control.

### Electroretinogram analysis

Electroretinograms (ERGs) were performed on mice as previously described with the Celeris system (Diagnosys LLC, Lowell, MA) and their preset programs for scotopic and photopic ERGs.^32^ In brief, mice were dark-adapted overnight for scotopic ERGs and anesthetized by an intraperitoneal injection of a ketamine/xylazine (100 and 10 mg/kg) mixture. One drop of each phenylephrine (2.5%) and tropicamide (1%) was applied for pupil dilation 10 min prior to recording. Animals were kept on a warming plate during the entire ERG procedure to maintain the body temperature at 37°C. After the scotopic recording, eyes were light-adapted for 10 min. The data shown represent the average of 6–7 mice at 2 months of age and were recorded with the following parameters: scotopic recordings used 1 cd·s/m^2^, whereas photopic recordings used a background intensity of 9 cd·s/m^2^ and a flash intensity of 10 cd·s/m^2^. In aged monkeys, central cone function was assessed by multifocal ERGs recorded with a VERIS system (Electro-Diagnostic Imaging, Milpitas, CA). Anesthesia was induced by intramuscular injection of 10 mg/kg ketamine, 1 mg/kg xylazine, and 0.4 mg atropine and maintained with subsequent partial doses as required. Supplemental oxygen was delivered by nasal cannula at 0.5 L/min, core body temperature was maintained between 37.0°C and 38.8°C by water-circulating heated pads, and heart rate and O_2_ saturation were monitored by pulse oximetry. Pupils were fully dilated (>8 mm) with 2–3 applications of tropicamide (1%) and phenylephrine (2.5%). The cornea was anesthetized with proparacaine (1%) and lubricated with methylcellulose (1%) before insertion of a bipolar Burian-Allen electrode, a subdermal needle electrode placed in the back served as ground. The electrodes were fitted with a +3 D contact lens to bring the stimulus display into approximate focus at the 40-cm stimulus distance. Mean screen luminance was 100 cd/m^2^, and field size was approximately 40°. Before mfERG data were recorded, the macula was aligned with the central stimulus hexagon using a reverse ophthalmoscope. Subsequent 1- or 2-min trial recordings were used to refine alignment such that the foveal response was centered in the mfERG response array. Eight-minute recordings were obtained from each eye using dynamic stimuli with both 103 and 241 unscaled hexagon elements.

### Fundus, optical coherence tomography, and angiography

Mouse fundus images were performed as previously described.^32^ Mice were anesthetized and eyes dilated as described for the ERG procedure. Image-guided OCT was used to take a 2D retinal cross-section at the sites where GA was suspected to confirm actual damage. All images were acquired with the Micron IV System from the Phoenix Technology Group (Lakewood, CO). Angiography was performed following fundus imaging by subcutaneously injecting 125 mg/kg of a fluorescein sodium solution. For retinal imaging of aged macaques, anesthesia was induced by an intramuscular injection of Telazol (1:1 mixture of tiletamine hydrochloride and zolazepam hydrochloride, 3.5–5.0 mg/kg), followed by intubation and maintenance with isoflurane (1%–2.5%) in 100% oxygen. Imaging modalities included color fundus photography (FF450; Zeiss, Oberkochen, Germany), spectral domain optical coherence tomography, and fundus autofluorescence (OCT/FAF; Heidelberg Spectralis, Heidelberg, Germany). The OCT scan pattern used a size of 30° × 25° with 61 B-scans and a distance between B-Scans of 109 μm.

### Drusen quantification in NHP

Heidelberg Eye Explorer (HEYEX) software was used to analyze drusen height on OCT images that were acquired as described above. The average height for each druse was obtained by averaging the thickness measured in every section in which the druse in question appeared, with thickness defined as the distance between the Choroid/BrM boundary and the RPE/POS boundary. To quantify drusen thickness, the thickness at each section that had a druse was subtracted from the average thickness of the two adjacent sections (first section prior to a druse and first section after a druse), as determined for each individual druse. To examine changes in drusen over time, we performed three types of analyses. In Figure 5B, we show the absolute changes in total drusen number from baseline (27 drusen for both OD eyes and 25 drusen for both OS eyes). For a druse to be counted at any given time point in this graph, it needed to be a clear visible druse with a distortion of the RPE/POS boundary. In Figures 5C and 5D, we tracked each individual druse over time to show the average drusen height change (Figure 5C) as well as the druse height to baseline ratio (Figure 5D). The ratio was calculated by dividing the average drusen height for each druse at each time point to its height at baseline prior to injection. Every location that had a druse at one time point was measured, even if at other time points no druse was present. If the baseline height was 0 (the druse appeared during the study), the number was set to 1 to avoid division by 0. If the druse height at any later time point was 0 (the druse disappeared or had not appear yet), then 0 was an acceptable value to record. A number 1 at baseline and 0 at 3 months would still result in a ratio of 0 at 3 months, meaning no druse present at baseline or 3 months at the specific location in question. Some thickness changes yielded small values rather than 0 when the druse was either not yet present or no longer present. However, these values resulted in ratios smaller than 1 and were used to track the overall behavior of all data points. The bar graphs in Figure 5D show the ratio for each individual druse as a dot and the average values as bars. A ratio = 1 indicates no change in height; a ratio greater than 1 indicates that the druse height increased, and a ratio smaller than 1 indicates that the druse height decreased. The average drusen height change shown in Figure 5C was calculated by subtracting the average of the absolute drusen heights at each time point from the average of the absolute drusen heights at baseline. The number was either positive if the average drusen heights increased or negative if the average drusen heights decreased. In contrast to the ratio, which provides information regarding the direction of change of each druse, the average drusen heights provide information on the absolute change in height.

### Histology

All retinal cross-sections, sample preparations, and immunostainings were performed as previously described.^32^^,^^95^ All animal eyes were cryopreserved in optical cutting temperature compound and sectioned at 12 μm thickness. In mice, the entire posterior part of the eye cup, including sclera, choroid/RPE, and retina, was collected. In both pigs and NHPs, the anterior part of the eyes, including cornea, lens, and vitreous, was removed. For each eye, a central band around 10 mm wide in the temporal nasal direction, including the macula in NHPs, was preserved for cryosections as shown in Figure 4. For human donor eyes, the cornea and lens were removed, and the remaining eye globes were preserved. Like NHP and pig retina, human donor eyes were sectioned along the nasal-temporal axis. Pig, NHP, and human tissues for sectioning were fixed for at least 24 h in 4% paraformaldehyde at 4°C. All antibodies used for histology are listed in Table S2 and were all diluted in PBS with 0.3% Triton X-100 and 5% bovine serum albumin (BSA, Cell Signaling Technology), except for the rabbit anti-pS6 (Ser240/244) antibody where PBS was replaced with TBS and the rabbit anti-apolipoprotein B (APOB), the goat anti-Apolipoprotein E (APOE), the rabbit anti-CFH, and goat anti-mouse complement C3 where Triton X-100 was replaced with 0.2% Saponin. Detection of signals used either fluorescent secondary antibodies (1:500, raised in donkey) that were purchased from Jackson Immuno Research and were purified F(ab)2 fragments that displayed minimal cross-reactivity with other species or an HRP-coupled secondary antibody that was purchased from Jackson Immuno Research (Cat. #: 711-036-152; 1:500) in conjunction with the immunohistochemistry staining kit ImmPACT VIP Kit (Vector Laboratories, Cat# SK-4605) as shown in (Figure 1). Fluorescent sections were counterstained with 4′, 6-diamidino-2-phenylindole (DAPI) (Sigma-Aldrich, Cat# 9542) to visualize nuclei. The following reagents already had a chromophore conjugated: rhodamine phalloidin (Life Technology, Cat. #: R415; 1:100) and fluorescein peanut agglutinin lectin (PNA; Vector Laboratories, Cat. #: FL-1071; 1:500). Expression changes for ApoB, ApoE, C3, and CFH were confirmed in at least three individual animals per genotype. For all data where signal intensities of antibody stainings were compared for qualitative purposes between different genotypes, disease, or treatment conditions, the staining was done in parallel with the samples being compared (Figures 1, 2C, 3A, 3F, 4B, S2C, S4D, S6B, S6C, S8, S9A, S9C, and S10). The identical batches of solutions, antibody dilutions, and incubation times were used, including the incubation time for the HRP reaction in Figure 1 to visualize pS6 signal. All histological images were acquired with a Leica DM6 Thunder microscope with a 16-bit monochrome camera. Primary antibodies used are listed in Table S2 of the supplemental information file.

### RNAscope

Tetra-siRNA^*S6k1*^ distribution was visualized by miRNAscope HD (RED) Assay (ACD Bio, Cat# 324531) with a probe specific to the siRNA sequence for *S6k1*. Standard retinal sections collected for antibody staining were processed according to the manufacturer’s instructions. Sections were counterstained with hematoxylin before mounting them in Histomount. In the mouse studies, three retinas from different mice were used to confirm distribution and longevity of the siRNA. For the rhesus macaques, only one eye was used to assess distribution at 1 month post-injection.

### Analysis of POS clearance by the RPE

Quantification of POS clearance was performed as previously described.^32^ Antibody staining was performed as previously described.^95^ For each group, 4–8 RPE flat mounts were analyzed. For each RPE flat mount, 10 areas of 40,000 μm^2^ within a 1.5 mm radius from the center were selected randomly to quantify the number of RHODOPSIN-positive dots per RPE cell. Images for quantification were acquired at 20× magnification. RPE cell boundaries were detected with an anti-ZO1 antibody. Quantification was performed using IMARIS imaging processor by selecting a dot diameter >2 μm to count dots and by counting the number of RPE cells per imaged field. The average dot number per RPE cell for a given RPE flat mount was obtained by averaging the results of the 10 fields. This number was then used to generate the average of the biological replicates, as indicated in the individual figures, per genotype and time point. Data are shown as the percentage of the ratio of POSs (RHODOPSIN positive dots) detected at 11 am versus the number detected at 8 am. Mice were analyzed at 2 months of age, as there are no other phenotypes seen at that age, meaning the effect is a direct consequence of activated mTORC1 in rods. Clearance of the POS in the mice injected with the tetra-siRNA^*S6k1*^ (Figure 3D) was done at 4 months of age to allow enough time for the siRNA to reach peak silencing and for the changes in POS phospholipids to occur and possibly influence the RPE phagolysosomal activity.

### PR-cell-specific silencing

The Ai9 Cre reporter line was crossed into the into ^*rod*^*Tsc1*^−/−^ mice to allow for expression of tdTomato in rod PR cells. For both tetra-siRNA^*S6k1*^ and tetra-siRNA^*NTC*^ groups, three mice were injected. Two months post-intravitreal injection, the two retinas from each animal were pooled as one biological sample and dissociated into single cells using papain according to the manufacturer’s instructions (Cat. #: 9035-81-1, Worthington Biochemical, Freehold, NJ). tdTomato-positive and -negative cells were separated by FACS. A small fraction of each sample was used to test the purity of the tdTomato-positive and -negative cells by performing antibody staining on dissociated cells with cell-type-specific markers (Figure S7; antibodies used are listed in Table S2). The remaining samples were used to measure S6K1 protein levels by ELISA (Cell Signaling, Cat. #: 7038C). b-actin ELISA was used to normalize the cell numbers from each FACS (Cell Signaling, Cat. #: 7880). Data shown are normalized to the tetra-siRNA^*NTC*^ group within the same batch of the FACS experiment.

### Lactate assay

A lactate assay (L-Lactate Assay kit, Abcam, Cat. # ab65330) was performed using six biological samples from 2-month-old mice, each composed of one retina from different animals. Each biological measurement was performed in triplicate. Retinas were dissected in ice-cold PBS and processed according to manufacturer’s instructions.

### Quantitative western blot analysis

Western blot analyses were done as previously described.^32^ In brief, enucleated eyes were dissected in cold PBS buffer. Dissected retinas (or RPE/Choroid) were immediately transferred into RIPA buffer (Thermo Fisher Scientific, Cat. #: 89900) with protease and phosphatase inhibitors (Thermo Fisher Scientific; Cat. #: 1861281) and homogenized by sonication. After 10-min centrifugation at 4°C at 13,000 RPM, protein extracts were transferred into a fresh tube, and protein concentration was quantified with the Bio-Rad Protein Assay (Cat. #: 500-0113,0114,0115). To quantify S6K1 and p-S6 expression levels, 10 μg of total retinal protein extracts were loaded on a precast protein gels from Bio-Rad (Cat. #: 4561093). For quantification of APOE expression levels, 10 μg of total RPE/choroid extracts were loaded onto the gel. Protein detection used fluorescently labeled secondary (1:10,000) antibodies from Licor in combination with the Odyssey system. Quantification was performed with Image Studio software. The combination of these tools allows for optimal linear quantification independent of any post-acquisition image modifications for visualization purposes. Each biological sample contained one retina or one RPE/choroid. Number of replicates are indicated as dots in each bar graph. Primary antibodies used are listed in Table S1 of the supplemental information file.

### Lipid profiling

Lipid profiling was performed as previously described.^32^^,^^96^ Each analysis group consisted of six retinas collected from six different animals. Briefly, tissue was homogenized in 40% aqueous methanol and then diluted to a concentration of 1:40, with 2-propanol/methanol/chloroform (4:2:1 v/v/vol) containing 20 mM ammonium formate and 1.0 μM PC (14:0/14:0), 1.0 μM PE (14:0/14:0), and 0.33 μM PS (14:0/14:0) as internal standards. Samples were introduced into a triple-quadrupole mass spectrometer (TSQ Ultra, Thermo Fisher Scientific) by using a chip-based nano-ESI source (Advion NanoMate) operating in infusion mode. PC lipids were measured using precursor ion scanning of *m/z* 184, PE lipids were measured using neutral loss scanning of *m/z* 141, and PS lipids were measured using neutral loss scanning of *m/z* 185. All species detected for each group are represented as a relative percentage of the sum based on their response values. Abundances of lipid molecular species were calculated using the Lipid Mass Spectrum Analysis (LIMSA) software (University of Helsinki, Helsinki, Finland). Lipid profiling for Figures 4D and 4E was performed by *Caymen Chemicals* on a Sciex ExionLC Integrated System.

### Oligonucleotide synthesis

Oligonucleotides were synthesized by phosphoramidite solid-phase synthesis on an AKTA Oligopilot 100 (Cytiva, Marlborough, MA) using 2ʹ-F or 2ʹ-O-Me modified phosphoramidites with standard protecting groups. 5'-(E)-Vinyl tetra phosphonate (pivaloyloxymethyl) 2′-O-methyl-uridine 3′-CE phosphoramidite (VP) was purchased from Hongene Biotech, USA. Tetravalent oligonucleotides were prepared using commercial doubler phosphoramidites as branching points (Glen Research, Sterling, VA) and tetraethyloxy-Glycol phosphoramidite (TEG) (ChemGenes, Wilmington, MA) as spacer from the solid support. Phosphoramidites were prepared at 0.1 M in anhydrous acetonitrile (ACN), except for 2′-O-methyl-uridine phosphoramidite dissolved in anhydrous ACN containing 15% dimethylformamide. 5-(Benzylthio)-1H-tetrazole (BTT) was used as the activator at 0.25 M, and the coupling time for all phosphoramidites was 4 min, except for doubler, and TEG phosphoramidites, where coupling time used was 8 min. Detritylation was performed using 3% trichloroacetic acid in Toluene (AKTA). Capping reagents used were CAP A (20% n-methylimidazole in ACN) and CAP B (20% acetic anhydride and 30% 2,6-lutidine in ACN). Phosphite oxidation to convert to phosphate or phosphorothioate was performed with 0.05 M iodine in pyridine-H2O (9:1, v/v) or 0.1 M solution of 3-[(dimethylaminomethylene)amino]-3H-1,2,4-dithiazole-5-thione (DDTT) in pyridine (ChemGenes) for 3 min. Reagents for detritylation, iodine oxidation, and capping were purchased from AIC. Oligonucleotides were synthesized on 1,000 Å long-chain alkyl amine (LCAA) controlled pore glass (CPG) functionalized with UnyLinker terminus (ChemGenes).

### Deprotection and purification of oligonucleotides for *in vivo* experiments

Multivalent (tetravalent) oligonucleotides were cleaved and deprotected in 28%–30% ammonium hydroxide-40% aq. methylamine (1:1, v/v) (AMA) for 2 h at room temperature. The VP containing oligonucleotides were cleaved and deprotected as described previously.^97^ Briefly, CPG containing VP oligonucleotides was treated with a solution of 3% diethylamine in 28%–30% ammonium hydroxide at 35°C for 20 h. All solutions containing cleaved oligonucleotides were filtered to remove the CPG and dried under vacuum. The resulting pellets were re-suspended in 5% ACN in water. Purifications were performed on an Agilent 1290 Infinity II HPLC system. VP and multivalent (tetravalent) oligonucleotides were purified using a 21.2 × 150 mm PRP-C18 column (Hamilton Co, Reno, NV); running conditions: eluent A, 400 mM hexafluoroisopropanol and 15 mM triethylamine in water; eluent B, 400 mM hexafluoroisopropanol and 15 mM triethylamine in methanol; linear gradient; 20%–38% B in 20 min at 60°C. VP and multivalent (tetravalent) oligonucleotides were buffer exchanged using a custom 20 × 150 mm column packed with Source 15Q anion exchange resin (Cytiva, Marlborough, MA); running conditions: eluent A, 20 mM tris base (pH 8.5) in 10% ACN in water; eluent B, 1 M sodium bromide in 20 mM tris base (pH 8.5) in 10% ACN in water; linear gradient, 18% to 45% B in 20 min at 50°C. Flow was 30 mL/min in both methods, and peaks were monitored at 260 nm for oligonucleotides. Fractions were analyzed by liquid chromatography-mass spectrometry (LC-MS); pure fractions were combined and dried under vacuum. Pure oligonucleotides were re-suspended in 5% ACN and desalted by size exclusion on 25 × 250 mm custom columns packed with Sephadex G-25 media (Cytiva, Marlborough, MA). Desalted oligonucleotides were finally lyophilized.

### Statistical analysis

Multiple *t* test analysis was used for two-group comparisons, and one-way ANOVA was used for multiple group comparisons. Two-tailed analyses were used in both analysis types. Significance levels are indicated as follows: ∗*p* < 0.05; ∗∗*p* < 0.01; ∗∗∗*p* < 0.001; and ∗∗∗∗*p* < 0.0001. Other parameters such as sample numbers, type of error bars, and averages are indicated in the figure legends.

## Data and code availability

All data and experimental parameters related to this paper are available in the main text and Supplemental Figures. All raw data from this study are available from the corresponding author upon reasonable request.

## Acknowledgments

We would like to thank Sara Kozma, Markus Rüegg, Michael Hall, and Ching-Kang Chen for mouse strains; Thomas Gallagher for critical reading of the manuscript; and Chih-Yun Cheng for the graphical abstract. This work was supported by a grant from the 10.13039/100006312BrightFocus Foundation (M2017071), 10.13039/100000053NEI grant R01EY032461, a pilot award from the UMass Center for Clinical and Translational Science, with funding from 10.13039/100006108NCATS award UL1TR001453, and a Bridge Fund award from the Innovation and Business Development Office of the 10.13039/100007920University of Massachusetts Chan Medical School to C.P. Other support includes a Macular Degeneration Center of Excellence Fund from the 10.13039/100007920University of Massachusetts Chan Medical School, Department of Ophthalmology and Visual Sciences and 10.13039/100000053NEI grant EY028602 to J.M.S.; an 10.13039/100000053NEI grant R01EY030513 to M.P.A.; a Research to Prevent Blindness unrestricted grant to the Dean McGee Eye Institute, Oklahoma City, Oklahoma; an ONPRC NIH Core grant P51OD011092; and 10.13039/100000002NIH grants S10OD020012 and R35GM131839 to A.K.

## Author contributions

S.-Y.C., D.G., S.K., J.M.S., J.C., K.G., N.McH., D.E., J.F.A., H.G.-E., H.R.B., L.R., H.W., J.S., T.J.M., R.S.B., M.-P.A., and C.P. performed experiments and interpreted the data. S.-Y.C., J.F.A., T.J.M., M.N., M.-P.A., A.K., and C.P. conceived the experiments and wrote the manuscript.

## Declaration of interests

Three provisional patent applications regarding the tetrameric chemistry used (63/412,051), the sequences used to target *S6k1* (63/412,092), and the use of *S6k1* as a therapeutic target for the treatment of AMD (63/013,395) have been submitted by the Innovation and Business Development Office of the University of Massachusetts Chan Medical School (authors in this publication: S.-Y.C., J.C., D.E., J.F.A., A.K., and C.P.). The authors declare that they otherwise have no conflicts of interest with this work.

## Supplemental information

Document S1. Figures S1–S13 and Tables S1 and S2

Document S2. Article plus supplemental information
