# Supplementary material for: An siRNA targeting S6k1 identifies photoreceptor phospholipid metabolism as a contributor to lipid buildup in age-related macular degeneration
Source: Mol Ther Nucleic Acids. 2026 Feb 28;37(2):102878. doi: 10.1016/j.omtn.2026.102878 (PMC12996783; doi:10.1016/j.omtn.2026.102878)
Supplement: Document S1. Figures S1–S13 and Tables S1 and S2 [file mmc1.pdf]

OMTN, Volume *37*

Supplemental information

An siRNA targeting *S6k1* identifies photoreceptor phospholipid metabolism as a contributor to lipid buildup in age-related macular degeneration

Shun-Yun Cheng, Delaney Giguere, San Kim, Johanna M. Seddon, Jillian Caiazzi, Katherine Gross, Nicholas McHugh, Dimas Echeverria, Julia F. Alterman, Heather Gray-Edwards, Hector Ribeiro Benatti, Lauren Renner, Hannah Woolard, Jonathan Stoddard, Trevor J. McGill, Martha Neuringer, Richard S. Brush, Martin-Paul Agbaga, Anastasia Khvorova, and Claudio Punzo

Table S1. Description of mouse models and crosses generated.

Mouse models	***Description***	***Phenotype***
$Tsc1^{fl/fl}$	Conditional allele for *Tsc1* gene, which gene product is Hamartin.	No phenotype unless crossed to cell-type specific Cre recombinase. Deletion results in constitutive activation of the kinase complex mTORC1.
$Rictor^{fl/fl}$	Conditional allele for *Rictor* gene: Rapamycin insensitive companion of MTOR Complex 2.	No phenotype unless crossed to cell-type specific Cre recombinase. Deletion results in loss of the kinase complex mTORC2.
iCre-75	Cre-recombinase expressed under the control of a 4kb promoter derived from the rod PR cell specific *rhodopsin* gene.	No phenotype unless crossed to a conditional allele. Results in gene loss of conditional allele in rod PR cells.
$S6k1^{-/-}$	Knockout mouse for Ribosomal protein S6 kinase B1 (*Rps6kb1*) also known as *S6k1*. *S6k2* can function as a redundant gene for some of the function of *S6k1*. *S6k2* is upregulated in many tissues in the absence of *S6k1*.	Mice are born smaller and grow less efficient that wild-type mice. No obvious eye phenotypes were observed in this study except for an increase in the scotopic a-wave amplitudes.

Genotypes Generated	***Description***	***Phenotype***	***Figures***	***Analysis Performed***
$^{rod}Tsc1^{-/-}$<br>$^{rod}Rictor^{-/-}$	Activation of mTORC1 and loss of mTORC2 in rod PR cells by combining the $Tsc1^{fl/fl}$, $Rictor^{fl/fl}$ and the *iCre-75* alleles into the same animals.	Causes AMD-like pathologies similar to activation of mTORC1 in rod PR cells.	Sup. Fig. 2	• Fundus<br>• OCT<br>• Angiography<br>• histology<br>• lactate assay
$^{rod}Tsc1^{-/-}$<br>$S6K1^{-/-}$	Activation of mTORC1 in rod PR cells and complete loss of *S6k1* in all cells by combining the $Tsc1^{fl/fl}$ and the *iCre-75* alleles with the *S6k1* knockout mouse alleles into the same animal.	Prevents the onset of any AMD like pathologies as previously described (PMID: 32434914). Heterozygous *S6k1* mice $^{rod}Tsc1^{-/-}$ $S6k1^{-/+}$ still develop pathologies albeit less frequent than mice with 2 copies of the wild-type *S6k1* gene ($^{rod}Tsc1^{-/-}$ $S6k1^{+/+}$).	Fig. 2<br>Sup. Fig. 3<br>Sup. Fig. 4	• Fundus<br>• OCT<br>• Angiography<br>• histology<br>• lactate assay<br>• ERG recordings<br>• POS phagocytosis assays<br>• PE(44:12) and PC(44:12) phospholipid profiling,
$^{rod}Tsc1^{-/-}$<br>tetra-siRNA^{S6k1}	Activation of mTORC1 in rod PR cells by combining the $Tsc1^{fl/fl}$ and the *iCre-75* allele into the same animals.	Causes AMD-like pathologies as previously described (PMID: 32434914) including Bruch's membrane lipid buildup, RPE atrophy and neovascular pathologies. The tetra-siRNA^{S6k1} injections in these mice alleviates pathologies.	Fig. 3<br>Sup. Fig. 6<br>Sup. Fig. 8<br>Fig. 4D,E	• histology<br>• POS phagocytosis<br>• phospholipid profiling,

Table S2. List of Antibodies (in alphabetical order)

Target	Vendor (Cat. #)	Use and Dilution
**APOB**	Abcam (ab20737)	Histology (1:300)
**APOE**	Millipore (178479)	Histology (1:1,000), Western Blot (1:1,000)
**b-actin**	Cell Signaling (31008)	Western Blot (1:1,000)
**Brn3a**	Synaptic System (411003)	Histology (1:300)
**Calbidin**	Abcam (ab229915)	Histology (1:300)
**CFH**	Antibodies-online (3023097)	Histology (1:300)
**Chx10**	Invitrogen (PA5-116119)	Histology (1:300)
**Cone Arrestin (CA)**	Millipore (ABI5282)	Histology (1:300)
**C3**	MP Biomedicals (55510)	Histology (1:300)
**GFAP**	Millipore (MAB5628)	Histology (1:500)
**Glutamine Synthetase**	Millipore (MAB302)	Histology (1:500)
**IBA-1**	FUJIFILM Wako (019-19741)	Histology (1:300)
**Prox1**	Abcam (ab199359)	Histology (1:300)
**RHODOPSIN**	Invitrogen (MAI-722)	Histology (1:300)
**RPE65**	Abcam (ab75936)	Histology (1:1,000)
**pS6 (S240/244)**	Cell Signaling (5364)	Histology (1:300), Western Blot (1:300),
**S6K1**	Cell Signaling (34475)	Histology (1:1,000), Western Blot (1:1,000)
**ZO-1**	ProteinTech (66452-1-Ig)	Histology (1:300)

Supplemental Figures

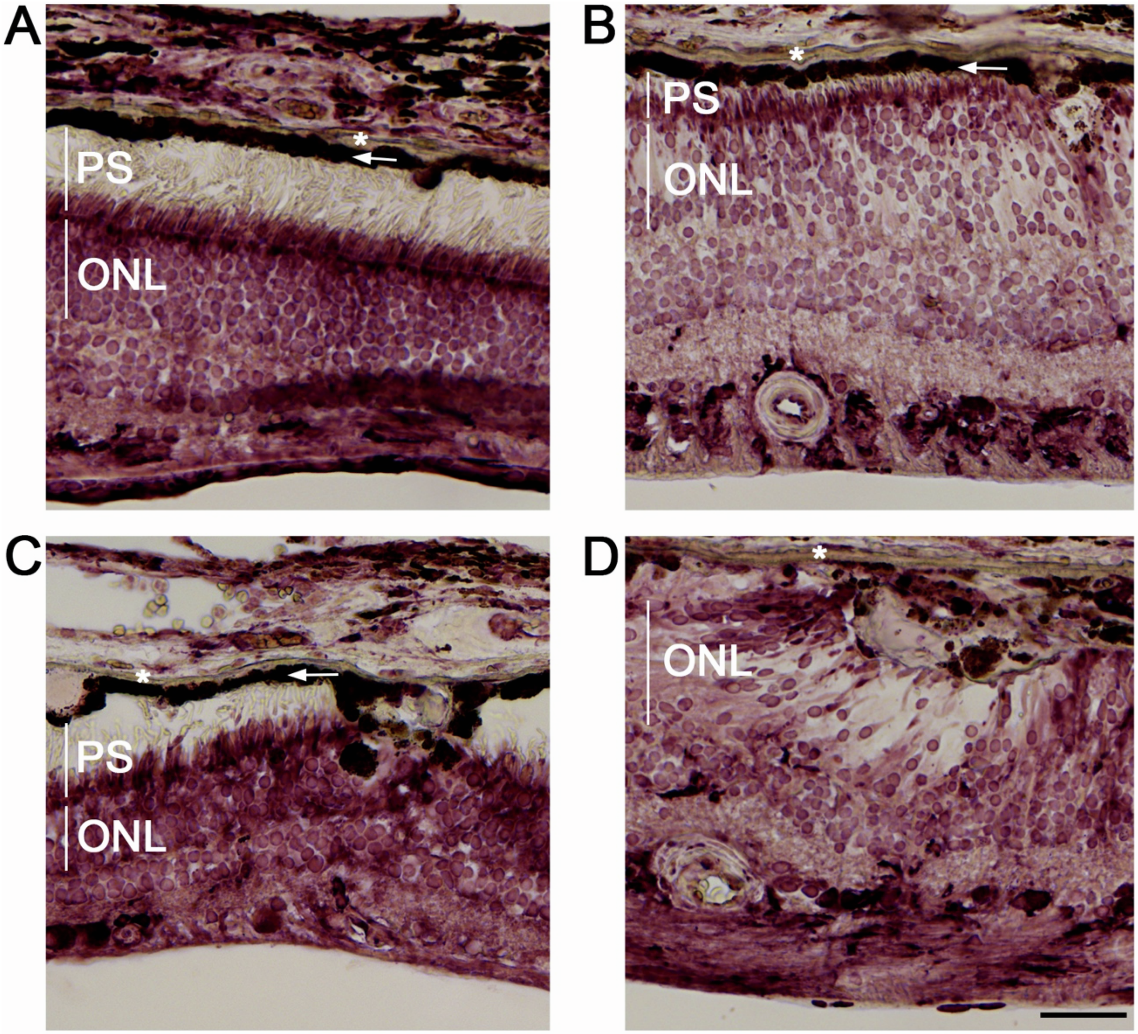

Figure S1. Phosphorylation of S6 across an entire retinal section. (A–D). Images from the same retinal cross-section of a 94-year (yrs)-old female (F) with wet AMD (grade 5B). (A-C) Central region (A), temporal region (B) and nasal region (C) of the same section showing PR cell inner and outer segments still present. The pigmented retinal-pigmented epithelium layer (white arrows) can be seen attached onto the Bruch's membrane (asterisks). (D) A different region located temporally with visible pathology. No clear retinal-pigmented epithelium layer is present at the Bruch's membrane. Photoreceptor inner and outer segments are mostly missing. Cross-section does not contain fovea. Scale bars = 50 µm; PS, marks PR segment region covering inner and outer segments; ONL, Outer nuclear layer; vertical bars mark thickness of ONL or PS in individual images. pS6 signal is shown in purple.

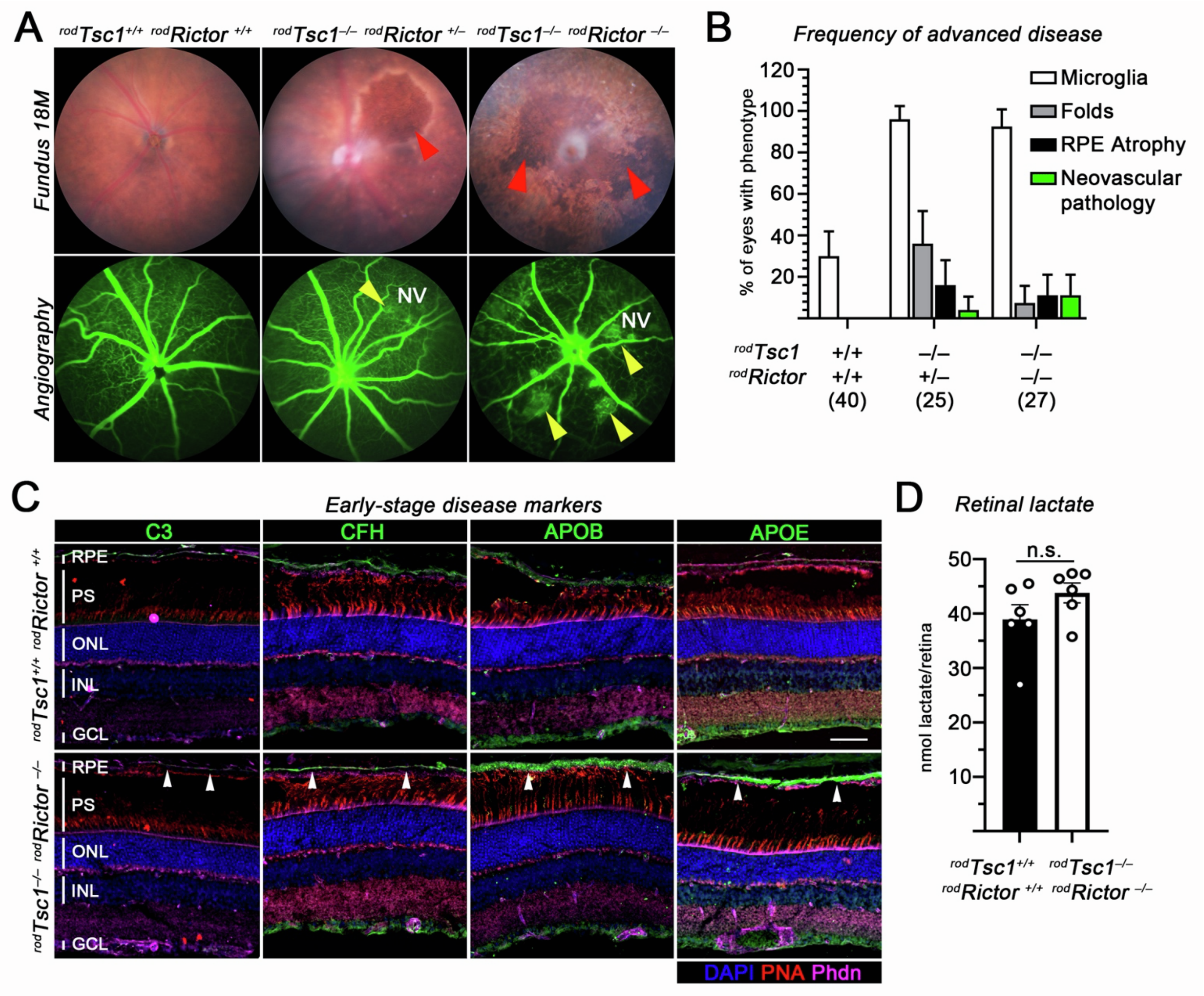

Figure S2. **mTORC2 activity in PRs modulates advanced pathologies.** (A) Fundus and fundus fluoresceine angiography (FFA) images of 18-month-old mice from indicated genotypes showing focal RPE atrophy (red arrowheads) and neovascular (NV) pathologies with blood leakages (yellow arrowheads). (B) Frequency of the disease phenotypes in 18-month-old mice of genotypes indicated with number of mice examined per genotype indicated in parenthesis. Error bar = M.O.E. (C) Retinal cross-sections of 12-month-old mice showing changes in early-stage disease markers including C3, CFH, APOB, and APOE (green signals). Compared to *Cre*$^{-}$ littermate controls, C3 accumulation is reduced and APOB and APOE accumulations are increased while CFH accumulation is similar to littermate controls at the RPE/BrM layer (white arrowheads) in ^{rod}*Tsc1*$^{-/-}$ ^{rod}*Rictor*$^{-/-}$ mice. Scale bar = 50 µm; blue, nuclear DAPI; red, peanut agglutinin lectin (PNA) marking cone PR segments; magenta, Pholloidin (Phdn) marking RPE boundaries; green, protein of interest indicated on top of each column; RPE, retinal-pigmented epithelium; PS, marks PR segment region covering inner and outer segments; ONL, outer nuclear layer; INL, inner nuclear layer; GCL, ganglion cell layer; vertical bars in sections mark the height of different layers. (D) Measurements of retinal lactate levels in 2-month-old mice of indicated genotypes (N = 6; n.s. = not significant; error bars = ± S.E.M.).

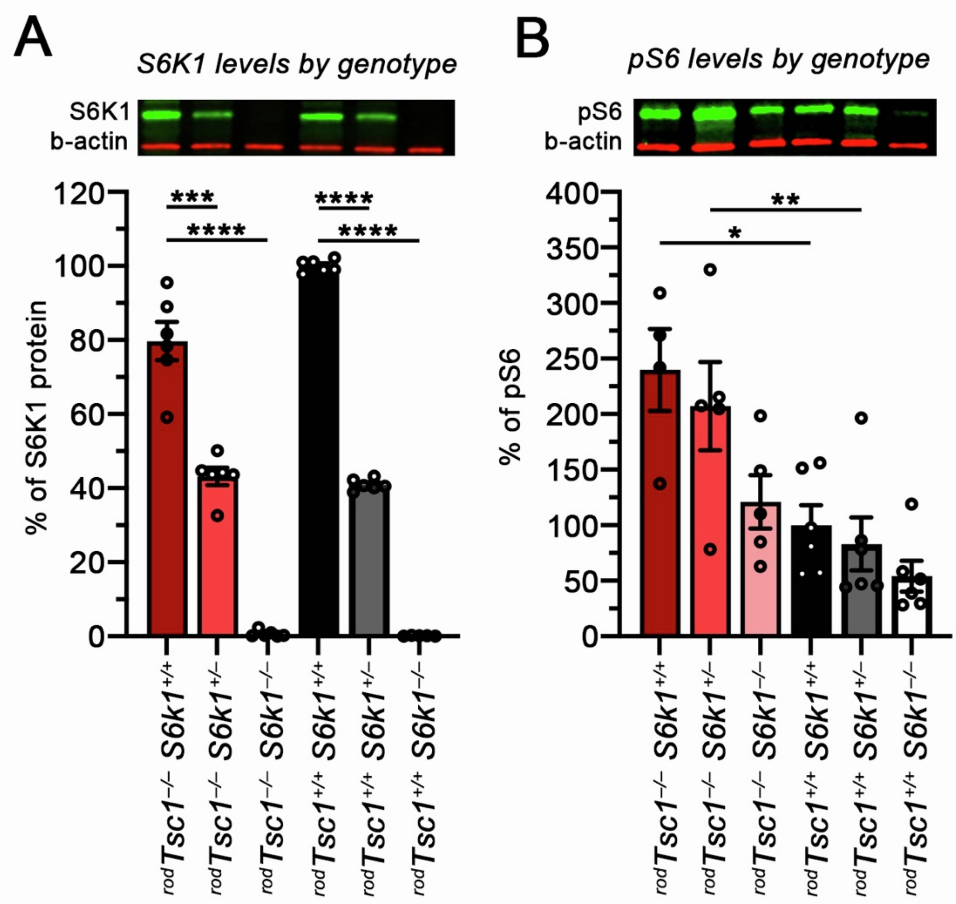

Figure S3. Confirmation of genotypes and allelic dose reduction of S6K1 levels. (A and B) Western blot analysis probing for S6K1 (A) or pS6(240/244) (B) levels. S6K1 protein levels are slightly decreased in $^{rod}Tsc1^{-/-}$ mice while pS6 level are the highest due to the constitutive activation of mTORC1 (N = 4–6 retinas from different mice at 2-months of age). Mice are littermate controls generated from heterozygous crosses and expression levels were normalized to the wild-type littermate $^{rod}Tsc1^{+/+}$ *S6k1*$^{+/+}$ mice. Representative image of the western blots with the proteins in question in green and the loading control (b-actin) in red is shown above each bar graph (error bars = ± S.E.M.; *p<0.05, **p< 0.01, ***p<0.001, ****p< 0.0001).

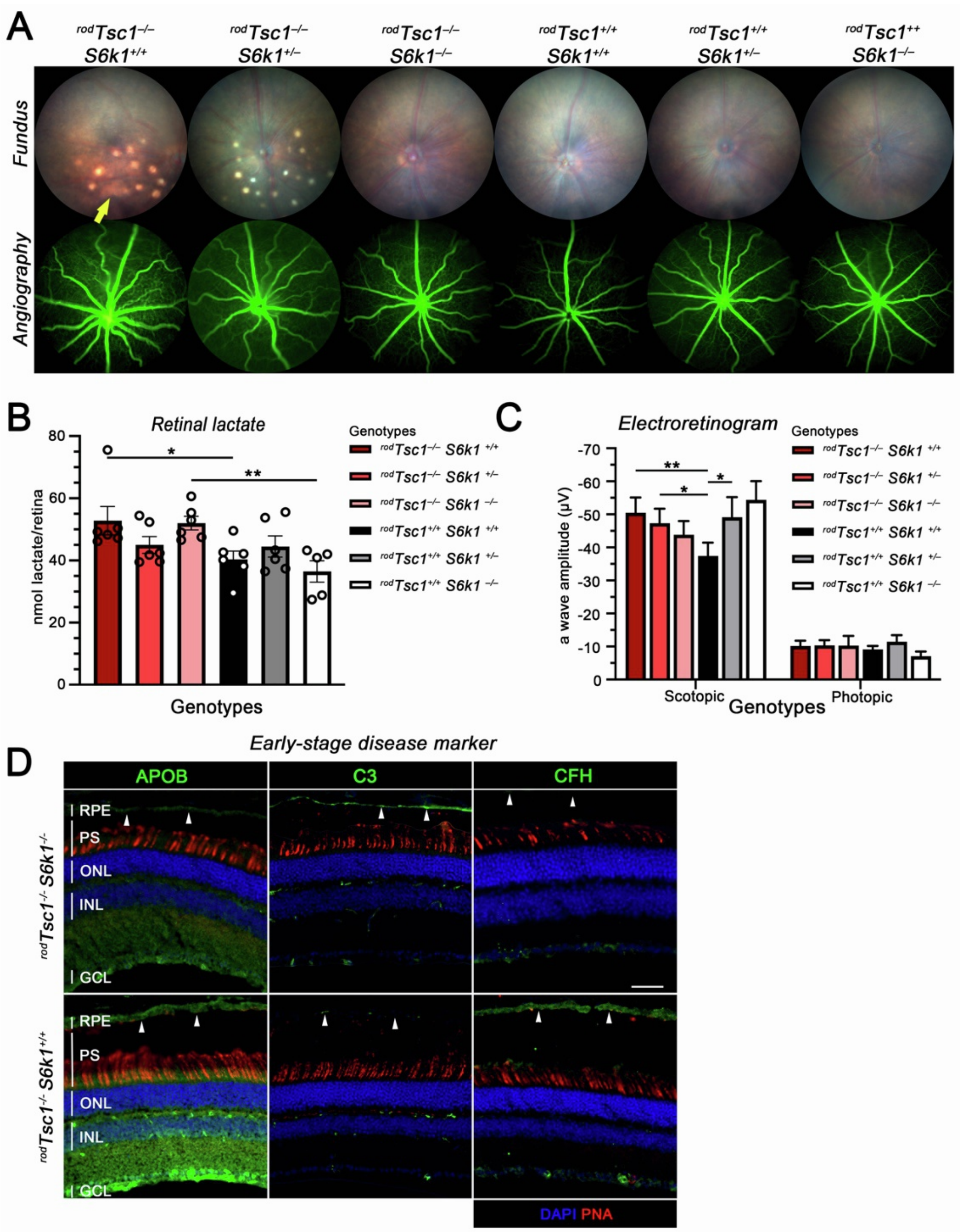

Figure S4. S6K1 activity is required for disease progression and onset in $^{rod}Tsc1^{-/-}$ mice. (A) Representative fundus and fluorescein angiography images of 12-month-old mice from indicated genotypes (GA, yellow arrow). (B) Lactate measurement from neural retinas of 2-month-old mice of genotypes indicated (N = 5–6 retinas from different mice; error bars = ± S.E.M.; *p<0.05, **p<0.01). (C) Electroretinogram recordings with 2-month-old mice of genotypes indicated showing the average scotopic (left bar graphs) and photopic (right bar graphs) a-wave amplitudes (N = 6–8 mice; error bars = ± S.E.M.; *p<0.05, **p<0.01). (D) Retinal cross-sections of 12-month-old mice showing early-stage disease markers (green signal) including APOB, C3, and CFH at the RPE/BrM (white arrowheads). There is almost no accumulation of APOB and CHF in $^{rod}Tsc1^{-/-}$ *S6k1*$^{-/-}$ mice, while C3 expression is restored. Scale bar = 50 µm; blue, nuclear DAPI; red, peanut agglutinin lectin (PNA) marking cone PR segments; green, protein of interest indicated on top of each column; RPE, retinal-pigmented epithelium; PS, marks PR segment region covering inner and outer segments; ONL, outer nuclear layer; INL, inner nuclear layer; GCL, ganglion cell layer; vertical bars in sections mark the height of different layers.

A

siRNA sequences used for screen

Duplex		Sequence
1	Sense	(mU)#(mA)#(mU)(mU)(fA)(fC)(fA)(mG)(fG)(mA)(mU)(mA)(mU)#(mU)#(mA)-TegChol
	anti-sense	P(mU)#(fA)#(mA)(mU)(mA)(fU)(mC)(mC)(mU)(mG)(mU)(mA)(mA)#(fU)#(mA)#(fG)#(mC)#(mU)#(mG)#(fA)
2	Sense	(mC)#(mA)#(mU)(mG)(fA)(fA)(fG)(mG)(fU)(mG)(mC)(mU)(mU)#(mA)#(mA)-TegChol
	anti-sense	P(mU)#(fU)#(mA)(mA)(mG)(fC)(mA)(mC)(mC)(mU)(mU)(mC)(mA)#(fU)#(mG)#(fG)#(mC)#(mA)#(mA)#(fA)
3	Sense	(mC)#(mA)#(mU)(mG)(fG)(fA)(fA)(mC)(fA)(mU)(mU)(mG)(mU)#(mG)#(mA)-TegChol
	anti-sense	P(mU)#(fC)#(mA)(mC)(mA)(fA)(mU)(mG)(mU)(mU)(mC)(mC)(mA)#(fU)#(mG)#(fC)#(mC)#(mA)#(mA)#(fG)
4	Sense	(mG)#(mA)#(mU)(mC)(fU)(fA)(fA)(mG)(fA)(mU)(mU)(mA)(mU)#(mU)#(mA)-TegChol
	anti-sense	P(mU)#(fA)#(mA)(mU)(mA)(fA)(mU)(mC)(mU)(mU)(mA)(mG)(mA)#(fU)#(mC)#(fU)#(mA)#(mG)#(mC)#(fC)
5	Sense	(mC)#(mA)#(mG)(mG)(fA)(fG)(fC)(mA)(fA)(mA)(mU)(mA)(mC)#(mU)#(mA)-TegChol
	anti-sense	P(mU)#(fA)#(mG)(mU)(mA)(fU)(mU)(mU)(mG)(mC)(mU)(mC)(mC)#(fU)#(mG)#(fU)#(mU)#(mA)#(mC)#(fU)
6	Sense	(mA)#(mA)#(mU)(mA)(fA)(fC)(fU)(mA)(fA)(mA)(mC)(mU)(mG)#(mG)#(mA)-TegChol
	anti-sense	P(mU)#(fC)#(mC)(mA)(mG)(fU)(mU)(mU)(mA)(mG)(mU)(mU)(mA)#(fU)#(mU)#(fC)#(mU)#(mG)#(mU)#(fA)
7	Sense	(mU)#(mA)#(mA)(mG)(fG)(fA)(fC)(mA)(fA)(mA)(mU)(mU)(mA)#(mG)#(mA)-TegChol
	anti-sense	P(mU)#(fC)#(mU)(mA)(mA)(fU)(mU)(mU)(mG)(mU)(mC)(mC)(mU)#(fU)#(mA)#(fG)#(mU)#(mU)#(mG)#(fC)
8	Sense	(mA)#(mA)#(mU)(mC)(fU)(fG)(fA)(mA)(fG)(mA)(mG)(mG)(mA)#(mU)#(mA)-TegChol
	anti-sense	P(mU)#(fA)#(mU)(mC)(mC)(fU)(mC)(mU)(mU)(mC)(mA)(mG)(mA)#(fU)#(mU)#(fG)#(mC)#(mA)#(mA)#(fC)
9	Sense	(mC)#(mA)#(mA)(mC)(fC)(fU)(fG)(mA)(fA)(mU)(mC)(mU)(mU)#(mU)#(mA)-TegChol
	anti-sense	P(mU)#(fA)#(mA)(mA)(mG)(fA)(mU)(mU)(mC)(mA)(mG)(mG)(mU)#(fU)#(mG)#(fU)#(mU)#(mU)#(mG)#(fC)
10	Sense	(mA)#(mA)#(mG)(mA)(fA)(fA)(fA)(mG)(fU)(mU)(mU)(mU)(mC)#(mA)#(mA)-TegChol
	anti-sense	P(mU)#(fU)#(mG)(mA)(mA)(fA)(mA)(mC)(mU)(mU)(mU)(mU)(mC)#(fU)#(mU)#(fU)#(mC)#(mA)#(mC)#(fA)
11	Sense	(mA)#(mA)#(mC)(mU)(fU)(fC)(fA)(mU)(fA)(mA)(mU)(mU)(mG)#(mU)#(mA)-TegChol
	anti-sense	P(mU)#(fA)#(mC)(mA)(mA)(fU)(mU)(mA)(mU)(mG)(mA)(mA)(mG)#(fU)#(mU)#(fC)#(mU)#(mC)#(mA)#(fA)
12	Sense	(mC)#(mA)#(mU)(mU)(fA)(fA)(fU)(mA)(fU)(mC)(mU)(mU)(mG)#(mA)#(mA)-TegChol
	anti-sense	P(mU)#(fU)#(mC)(mA)(mA)(fG)(mA)(mU)(mA)(mU)(mU)(mA)(mA)#(fU)#(mG)#(fC)#(mU)#(mA)#(mA)#(fG)
13	Sense	(mU)#(mU)#(mU)(mA)(fU)(fA)(fA)(mA)(fA)(mU)(mA)(mU)(mA)#(mC)#(mA)-TegChol
	anti-sense	P(mU)#(fG)#(mU)(mA)(mU)(fA)(mU)(mU)(mU)(mU)(mA)(mU)(mA)#(fA)#(mA)#(fG)#(mC)#(mU)#(mC)#(fU)
14	Sense	(mU)#(mU)#(mU)(mU)(fC)(fU)(fA)(mC)(fA)(mU)(mU)(mC)(mU)#(mG)#(mA)-TegChol
	anti-sense	P(mU)#(fC)#(mA)(mG)(mA)(fA)(mU)(mG)(mU)(mA)(mG)(mA)(mA)#(fA)#(mA)#(fU)#(mA)#(mA)#(mA)#(fA)
15	Sense	(mC)#(mU)#(mU)(mG)(fU)(fA)(fC)(mC)(fA)(mG)(mG)(mU)(mA)#(mA)#(mA)-TegChol
	anti-sense	P(mU)#(fU)#(mU)(mA)(mC)(fC)(mU)(mG)(mG)(mU)(mA)(mC)(mA)#(fA)#(mG)#(fC)#(mA)#(mU)#(mG)#(fA)
16	Sense	(mU)#(mC)#(mC)(mU)(fU)(fA)(fA)(mC)(fA)(mA)(mU)(mU)(mU)#(mU)#(mA)-TegChol
	anti-sense	P(mU)#(fA)#(mA)(mA)(mA)(fU)(mU)(mG)(mU)(mU)(mA)(mA)(mG)#(fG)#(mA)#(fA)#(mG)#(mG)#(mU)#(fA)
17	Sense	(mC)#(mA)#(mG)(mG)(fC)(fA)(fG)(mA)(fA)(mG)(mU)(mU)(mG)#(mU)#(mA)-TegChol
	anti-sense	P(mU)#(fA)#(mC)(mA)(mA)(fC)(mU)(mU)(mC)(mU)(mG)(mC)(mC)#(fU)#(mG)#(fU)#(mC)#(mC)#(mC)#(fA)
18	Sense	(mC)#(mA)#(mU)(mA)(fA)(fU)(fU)(mU)(fA)(mA)(mA)(mG)(mU)#(mG)#(mA)-TegChol
	anti-sense	P(mU)#(fC)#(mA)(mC)(mU)(fU)(mU)(mA)(mA)(mA)(mU)(mU)(mA)#(fU)#(mG)#(fC)#(mU)#(mU)#(mC)#(fA)
19	Sense	(mC)#(mA)#(mA)(mA)(fU)(fA)(fA)(mG)(fG)(mG)(mU)(mU)(mA)#(mU)#(mA)-TegChol
	anti-sense	P(mU)#(fA)#(mU)(mA)(mA)(fC)(mC)(mC)(mU)(mU)(mA)(mU)(mU)#(fU)#(mG)#(fA)#(mC)#(mA)#(mU)#(fC)
20	Sense	(mA)#(mC)#(mU)(mA)(fA)(fA)(fU)(mG)(fA)(mU)(mU)(mC)(mC)#(mU)#(mA)-TegChol
	anti-sense	P(mU)#(fA)#(mG)(mG)(mA)(fA)(mU)(mC)(mA)(mU)(mU)(mU)(mA)#(fG)#(mU)#(fG)#(mU)#(mA)#(mA)#(fA)
21	Sense	(mC)#(mA)#(mA)(mA)(fA)(fU)(fA)(mG)(fU)(mA)(mU)(mU)(mG)#(mU)#(mA)-TegChol
	anti-sense	P(mU)#(fA)#(mC)(mA)(mA)(fU)(mA)(mC)(mU)(mA)(mU)(mU)(mU)#(fU)#(mG)#(fC)#(mU)#(mU)#(mG)#(fA)

B

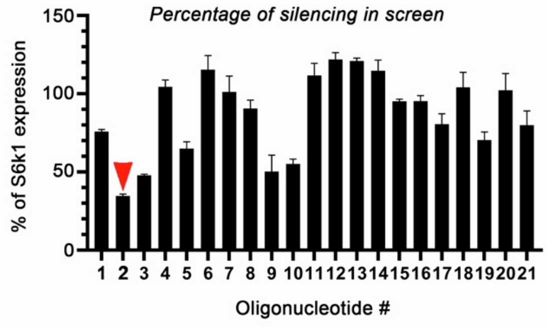

Figure S5. ***In vitro* screen of different siRNA sequences targeting *Rbs6k1b* in a mouse cell line.** (A) Sequences of the 21 different siRNAs used for the screen. (B) Silencing efficiencies of the different siRNAs was determined in N2A cells by measuring *S6k1* mRNA expression levels 3 days post treatment compared to untreated control cells. Each measurement was done in triplicate (N = 3; error bars = ± S.E.M.). X-axis indicates the oligo # and Y-axis the silencing efficiency. The guide siRNA with the strongest silencing efficiency (red arrowhead: ~60% silencing) was chosen for all subsequent *in vivo* experiments. The sequence is 100% conserved among mammals.

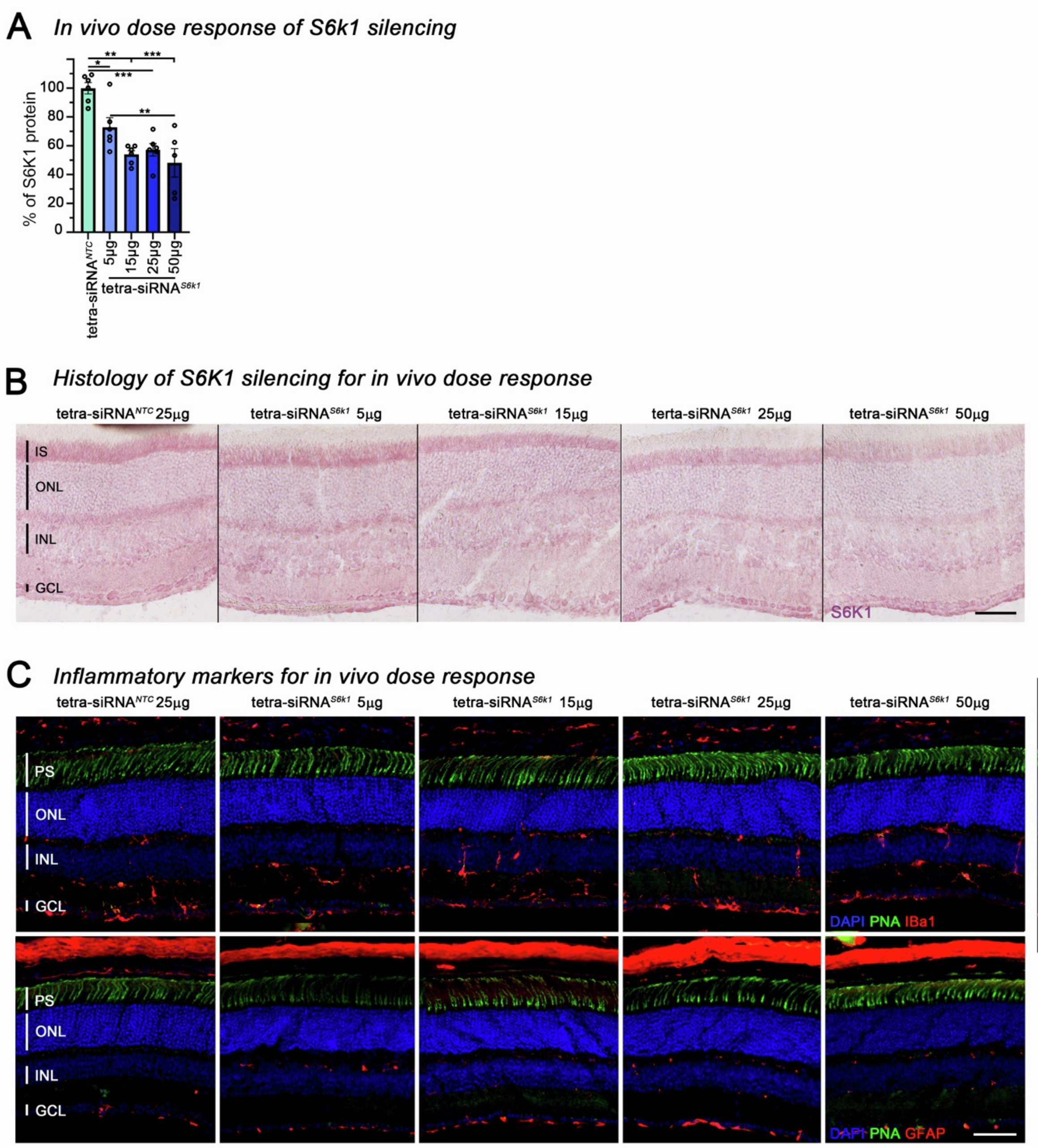

Figure S6. ***In vivo*** **dose response of tetra-siRNA**^{S6k1}**.** (A) Silencing efficiency in mouse 2 months after intravitreal delivery of the tetra-siRNA^{S6k1} at doses indicated. Average expression levels of S6K1 protein were adjusted to the NTC (non-targeting control) injections (N = 5–6 retinas). Silencing efficiency in mouse plateaued at around 50% for doses of 15 µg or more (error bars = ± S.E.M.; *p<0.05, **p<0.01, ***p<0.001). (B and C) Retinal cross-sections 2 months post intravitreal injection of the tetra-siRNA^{S6k1} at doses indicated on top of each panel. (B) Shown is the reduction of S6K1 protein (pink signal) particularly in the PR inner segment (IS) region and (C) the absence of expression changes in the inflammatory markers Iba1 and GFAP (red signal). Scale bars = 50 µm; blue, nuclear DAPI; green, peanut agglutinin lectin (PNA) marking cone PR segments; red, protein of interest indicated in panels of last column; PS, marks PR segment region covering inner and outer segments; ONL, outer nuclear layer; INL, inner nuclear layer; GCL, ganglion cell layer; vertical bars in sections mark the height of different layers.

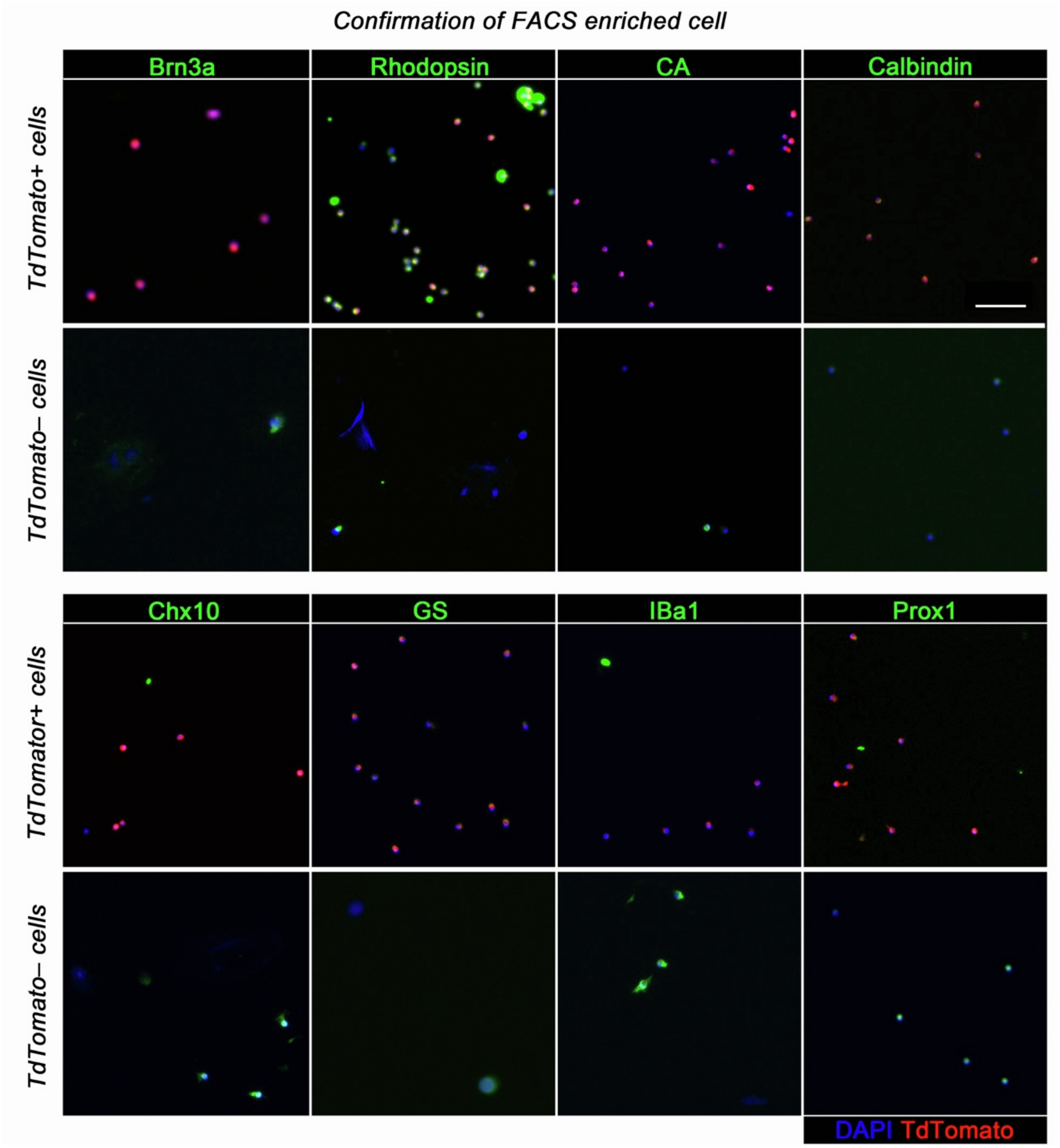

Figure S7. Confirmation of rod photoreceptor cell enrichment by FACS. Antibody staining of tdTomato+ (positive) and tdTomato– (negative) cells with different cell type-specific antibodies. The antibody and cell types are as follows: Brn3a, ganglion cells; Rhodopsin, rod photoreceptor cells; CA, cone arrestin, cone photoreceptor cells; Calbindin, horizontal cells and subtypes of amacrine and ganglion cells; Chx10, bipolar cells; GS, glutamine synthetase, Müller glia cells; Iba1, microglia; Prox1, amacrine and horizontal cells; all cell type specific antibodies are shown in green; blue, nuclear DAPI; red, tdTomato; Scale bar = 50 µm. Mice harboring the rod specific *iCre-75* and the Ai9 Cre-reporter (*loxP*-flanked STOP cassette followed by tdTomato) were injected with the tetra-siRNA^{S6k1} or the NTC. Two months post intravitreal injection retinas were dissociated and subjected to FACS to collect tdTomato positive and negative cells. Silencing efficiency of *S6k1* in tdTomato positive and negative cells was measured by protein ELISA for S6K1 to compare the tetra-siRNA^{S6k1} eyes to the NTC injected eyes as shown in (Figure 3C). The antibody stainings on the dissociated cells confirm that tdTomato positive cells are rod PR cells versus tdTomato negative cells, which are enriched for all other cell types.

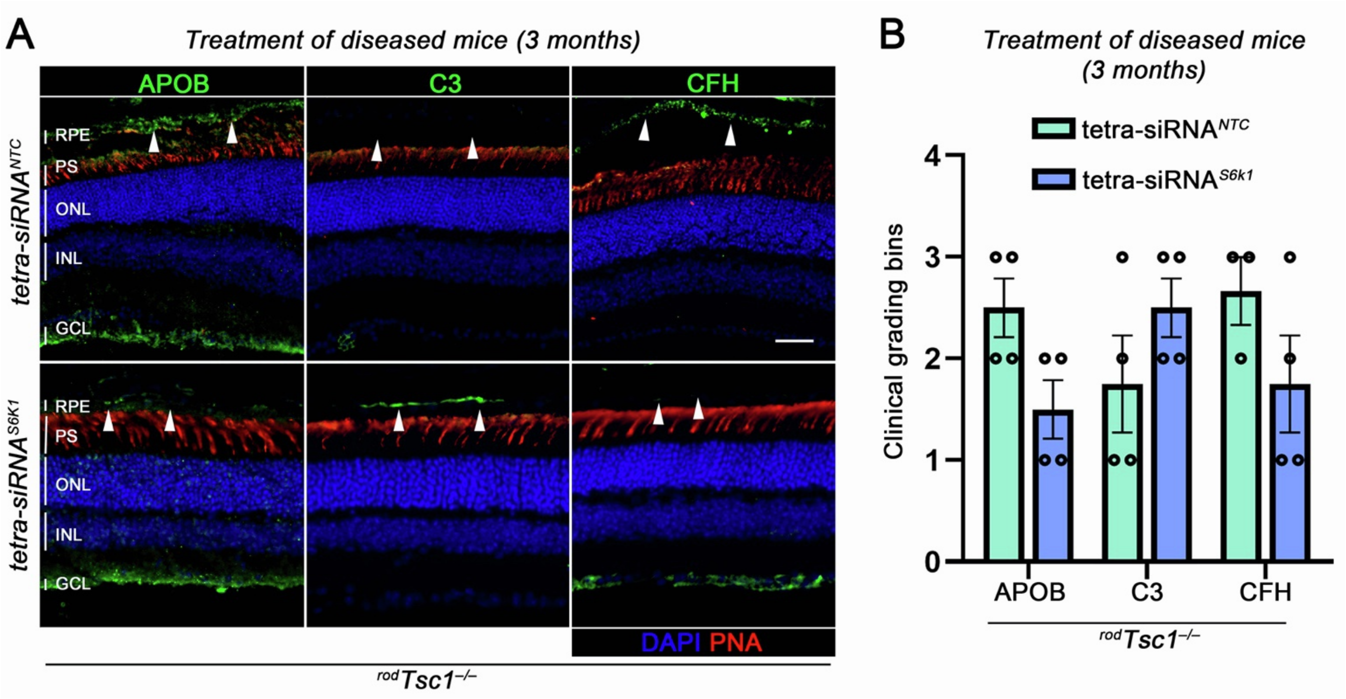

Figure S8. Silencing of *S6k1* reduces early disease pathologies. (A) Antibody stainings on retinal cross-sections of 15 months old mice treated at 12 months of age with one intravitreal injection of 15 µg of tetra-siRNA^{S6k1} or tetra-siRNA^{NTC}. Sections were stained for the early disease markers APOB, C3, and CFH (green signal). Eyes injected with the tetra-siRNA^{S6k1} (second row) show a clear reduction in APOB and CFH at the RPE/BrM and a restoration of C3 (white arrowheads mark RPE/BrM area) when compared to NTC injected eyes (first row). Blue, nuclear DAPI; red, peanut agglutinin lectin (PNA) marking cone segments; green, ApoB, C3 or CFH as indicated on top of columns; Scale bar = 50 µm; RPE, retinal-pigmented epithelium; PS, marks PR segment region covering inner and outer segments; ONL, outer nuclear layer; INL, inner nuclear layer; GCL, ganglion cell layer; vertical bars in section mark the height of different layers. (B) Bar graphs showing average from clinical grading of the histology shown in (A) by binning the staining in 4 different intensity bins. Each dot represents a central section from a different animal injected with the tetra-siRNA^{S6k1} or tetra-siRNA^{NTC}. Error bars = S.D.

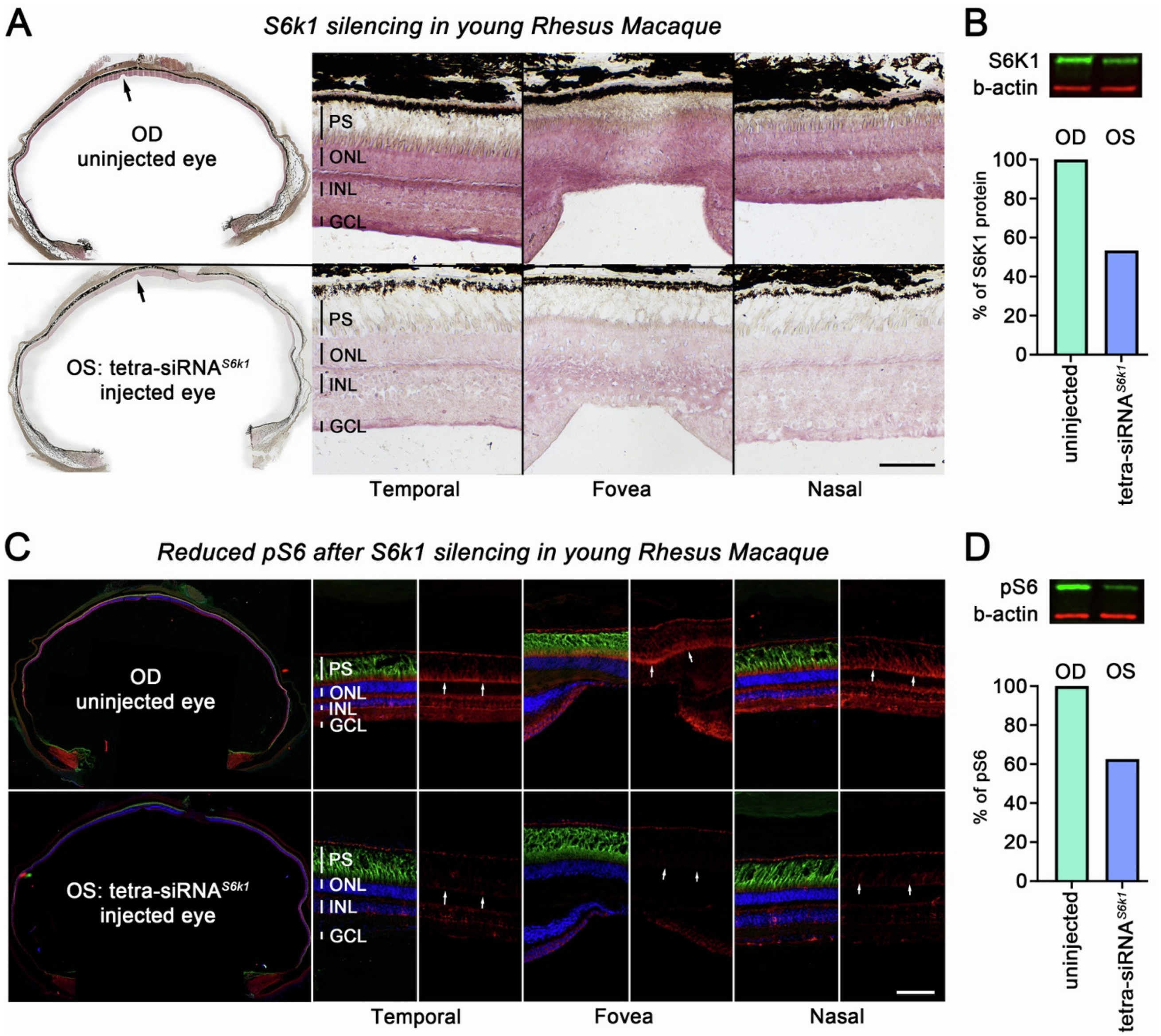

Figure S9. Silencing of *S6k1* in rhesus macaque. (A) Antibody staining for S6K1 on retinal cross-section of a rhesus macaque 1 month post intravitreal injection of 225 µg of the tetra-siRNA^{S6k1} into the left eye (OS, bottom row). The reduction of the purple signal between the uninjected (OD, right eye, top row) and injected eye (OS, left eye) as seen in the overview images (first column, fovea marked by arrow) as well as the higher magnification images (columns 2–4) of a temporal, central (fovea), and nasal region shows that the siRNA leads to a uniform silencing of *S6k1* across the eye. (B) Western blot analysis showing ~50% reduction of S6K1 protein levels in the treated eye. Gel image on top shows protein bands of interest including the loading control b-actin. (C) Phosphorylation of S6 (pS6, red signal) by immunofluorescence on sections from the same eye as used in (A). Red signal shows pS6 is reduced across the retina and in PR inner segments (arrows) of the treated eye. Blue, nuclear DAPI; green, peanut agglutinin lectin (PNA) marking cone segments; blue and green signals were removed from 50% of each panel to better visualize the red signals. (D) Western blot showing ~40% reduction in pS6. (A and C) Scale bars = 100 µm; PS, marks PR segment region covering inner and outer segments, ONL, outer nuclear layer; INL, inner nuclear layer; GCL, ganglion cell layer; vertical bars in section mark the height of different layers. (B) and (D) Error bars are omitted because these are results from N = 1 quadrant.

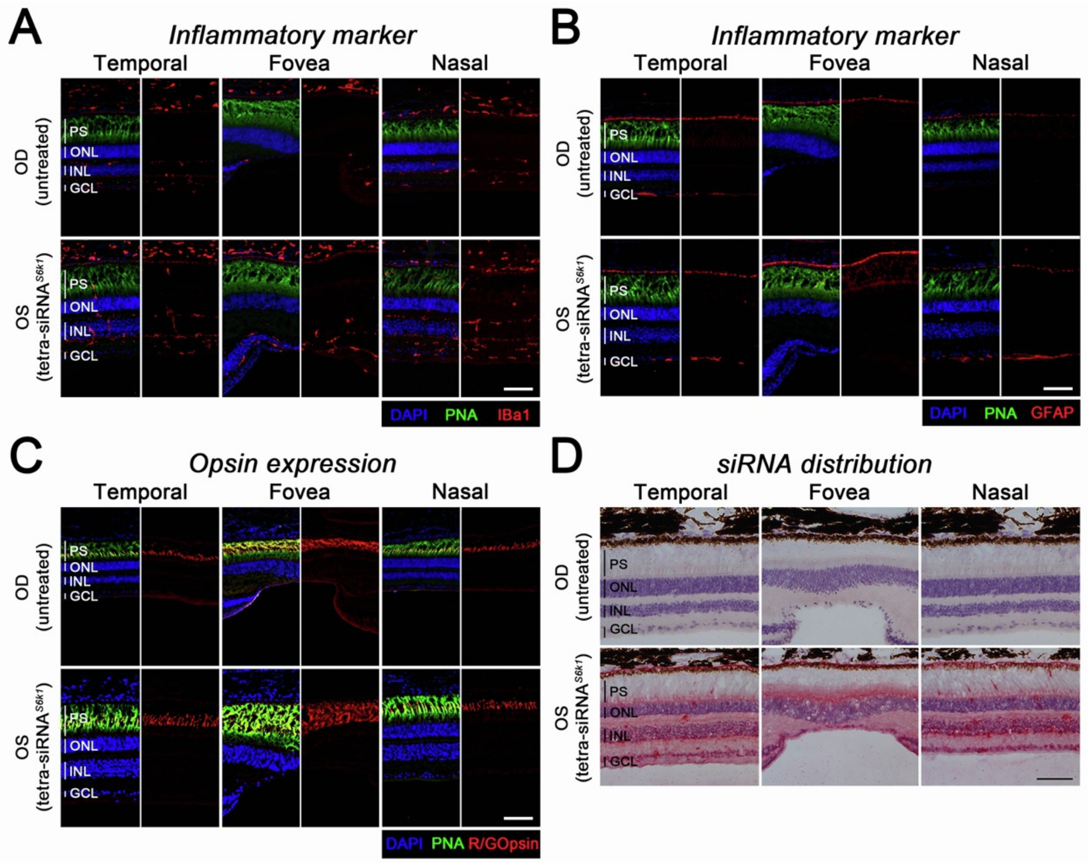

Figure S10. Safety of *S6k1* silencing in rhesus macaque. (A–C) Antibody staining for Iba1 (A), GFAP (B) and red/green opsin (C: R/GOpsin, aka: medium wave-length opsin marking cone PR cells) on retinal cross-section of a rhesus macaque 1 month post intravitreal injection of 225 µg of the tetra-siRNA^{S6k1} into the left eye (OS, bottom rows). Sections are from same animal as shown in Figure S9. Both inflammatory markers (Iba1, microglia; GFAP, reactive gliosis) show no increase in the injected eye (OS, left eye, second row) compared to the uninjected eye. (C) Expression of cone opsin remains normal in the injected eye (OS, left eye, second row). (A–C) Blue, nuclear DAPI; green, peanut agglutinin lectin (PNA) marking cone segments, red, Iba1, GFAP and R/GOpsin respectively; blue and green signals were removed from 50% of each panel to better visualize the red signals. (D) RNAscope showing the distribution of the tetra-siRNA^{S6k1} (red signal) across the various regions of the retina (purple signal represents nuclear counterstain with hematoxylin). (A–D) Scale bars = 100 µm; PS, marks PR segment region covering inner and outer segments, ONL, outer nuclear layer; INL, inner nuclear layer; GCL, ganglion cell layer; vertical bars in section mark the height of different layers.

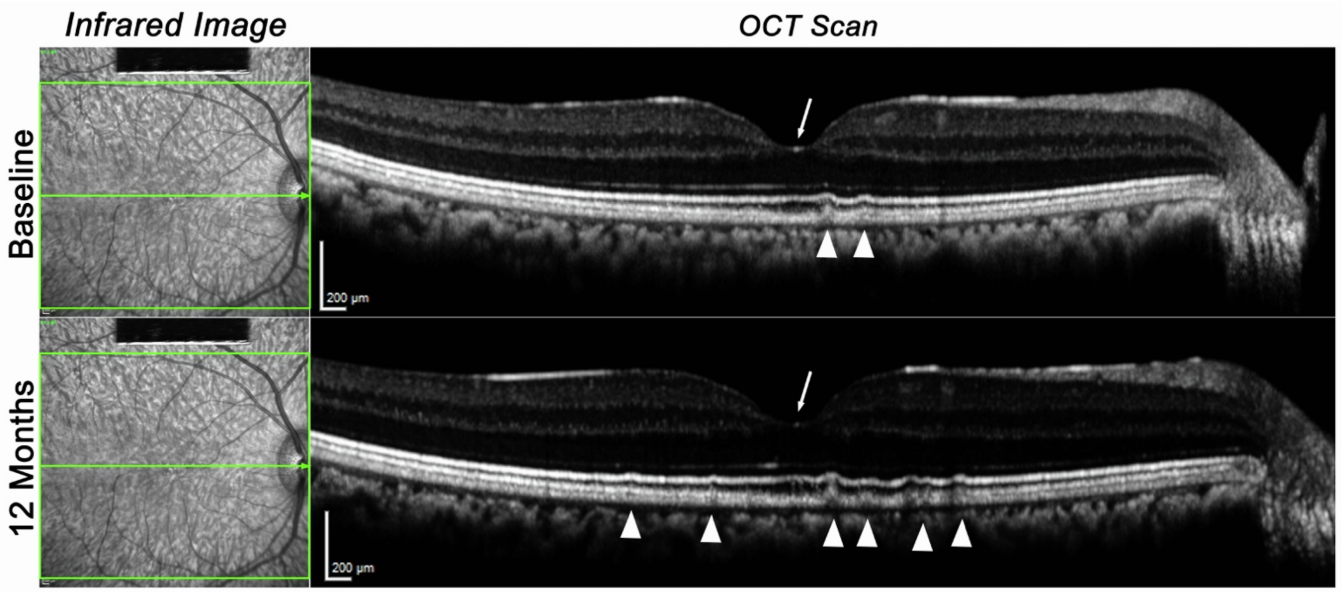

Figure S11. OCT scan region used to quantify drusen size. Shown is the right (OD) eye of the same rhesus macaques at baseline (top row) and 12-months post-injection (bottom row). On the left is a composite surface infrared image showing the area (green box) that was scanned during each follow-up examination and used for the quantifications presented (Figure 5). The raster scan included 61 optical sections within the green box. Each follow-up scan post-injection was aligned with the previous scan by an image-guided software that identifies the same area before generating the raster scan. The sections shown are both # 31 cutting through the plane of the fovea (white arrows). The sectioning plane is marked by the central green arrow in the infrared image on the left. Changes in the number and height of drusen (white arrowheads; two new ones of the left are small drusen that are just starting to develop, two new ones on the right are already more developed) were scored over time for all 61 sections by tracking every druse seen within the scanned area and its change in height or appearance and disappearance over time.

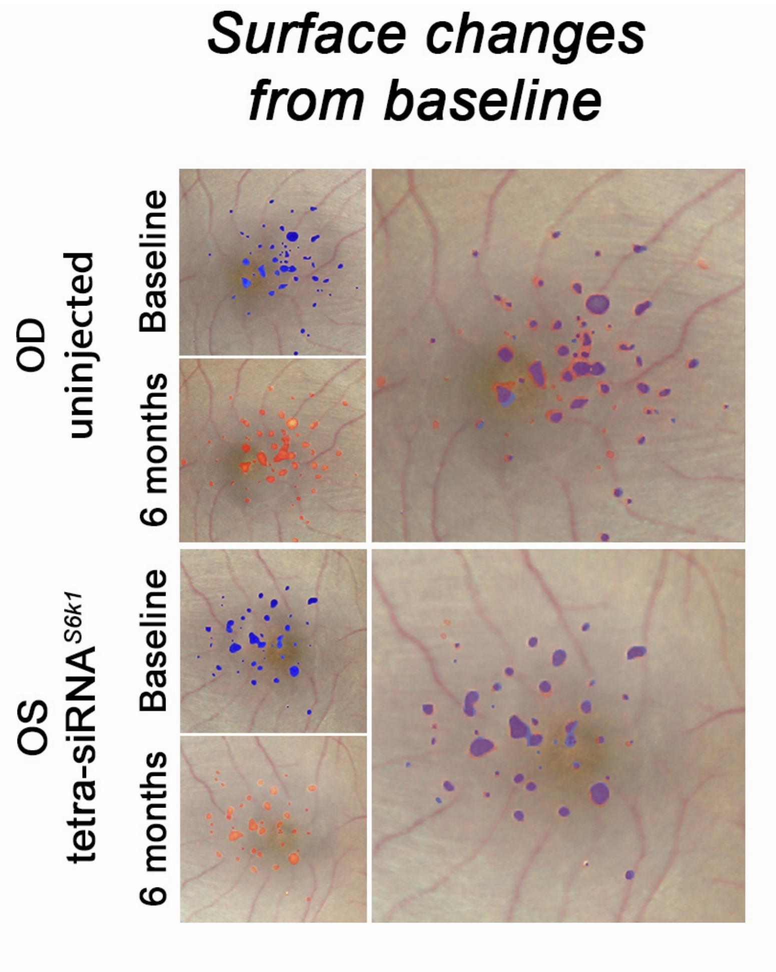

Figure S12. Changes in surface area covered by drusen. Images shown are the same as shown in Figure 5E showing difference between baseline and 6 months post-injection. Blue shows baseline drusen area and red shows area at 6 months. In the merged enlarged image, purple indicates no change while red indicates an increase in the surface area covered by drusen, showing that drusen appear relatively stable in the injected (OS) eye.

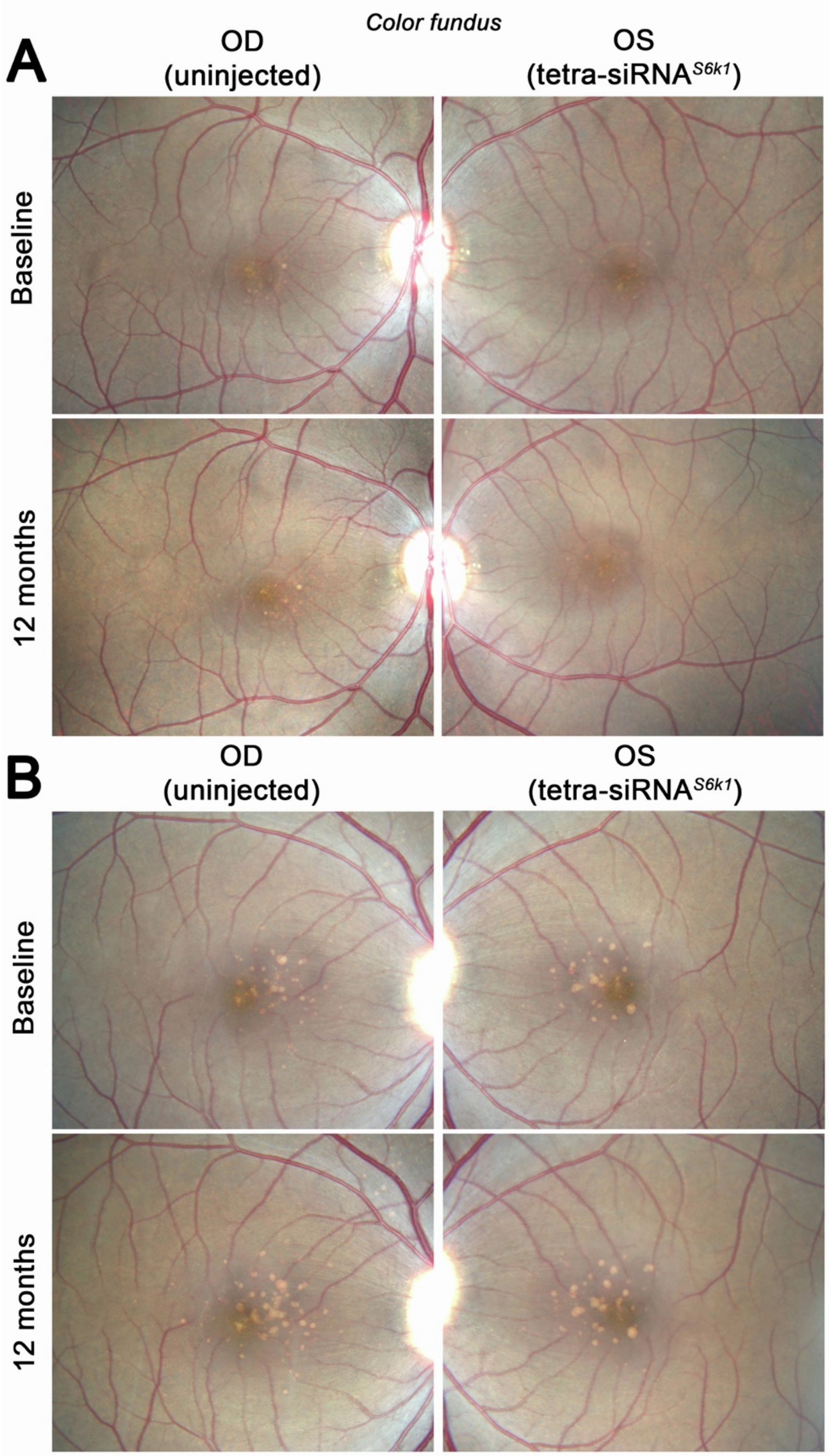

Figure S13. Color fundus images of treated rhesus macaques. (A) Color fundus photographs of the 21-year-old rhesus macaque, and (B) of the 22-year-old rhesus macaque treated with 225 µg of tetra-siRNA^{S6k1} in their left eyes (OS). Shown are fundus images at baseline and 12 months after the intravitreal injection. In both NHPs the treated left eye (OS) appears to have progressed less when compared to the untreated right eyes within the same NHP (OD: right eye, untreated; OS: left eye, treated).
